# MAPK pathway inhibition induces MET and GAB1 levels, priming BRAF mutant melanoma for rescue by hepatocyte growth factor

**DOI:** 10.18632/oncotarget.14855

**Published:** 2017-01-27

**Authors:** Sean Caenepeel, Keegan Cooke, Sarah Wadsworth, Guo Huang, Lidia Robert, Blanca Homet Moreno, Giulia Parisi, Elaina Cajulis, Richard Kendall, Pedro Beltran, Antoni Ribas, Angela Coxon, Paul E. Hughes

**Affiliations:** ^1^ Department of Oncology Research, Amgen, Inc., Thousand Oaks, CA, USA; ^2^ Department of Medicine, Division of Hematology-Oncology, David Geffen School of Medicine, University of California Los Angeles (UCLA), Los Angeles, CA, USA

**Keywords:** melanoma, resistance, BRAF, HGF, MET

## Abstract

Therapeutic resistance is a major obstacle to achieving durable clinical responses with targeted therapies, highlighting a need to elucidate the underlying mechanisms responsible for resistance and identify strategies to overcome this challenge. An emerging body of data implicates the tyrosine kinase MET in mediating resistance to BRAF inhibitors in BRAF^V600E^ mutant melanoma. In this study we observed a dominant role for the HGF/MET axis in mediating resistance to BRAF and MEK inhibitors in models of BRAF^V600E^ and NRAS mutant melanoma. In addition, we showed that MAPK pathway inhibition induced rapid increases in MET and GAB1 levels, providing novel mechanistic insight into how BRAF^V600E^ mutant melanoma is primed for HGF-mediated rescue. We also determined that tumor-derived HGF, not systemic HGF, may be required to convey resistance to BRAF inhibition *in vivo* and that resistance could be reversed following treatment with AMG 337, a selective MET inhibitor. In summary, these findings support the clinical evaluation of MET-directed targeted therapy to circumvent resistance to BRAF and MEK inhibitors in BRAF^V600E^ mutant melanoma. In addition, the induction of MET following treatment with BRAF and MEK inhibitors has the potential to serve as a predictive biomarker for identifying patients best suited for MET inhibitor combination therapy.

## INTRODUCTION

Approximately half of all malignant melanomas harbor an activating mutation in the serine/threonine kinase BRAF; ~90% of these mutations involve a valine to glutamic acid substitution at residue 600 (V600E) [1]. This mutation constitutively activates the mitogen-activated protein kinase (MAPK) signaling pathway driving proliferation and survival [1–3]. The BRAF inhibitor vemurafenib was the first approved targeted therapy for BRAF^V600E^ metastatic melanoma, achieving a response rate of 50% to 60% and significantly improving progression-free survival (PFS) and overall survival (OS) [4, 5]. More recently, combination therapy of BRAF and MEK inhibitors has improved response rates to approximately 70% in patients with BRAF^V600E^ mutant melanoma while exhibiting evidence of clinical benefit in almost all patients [6]. However, nearly all patients that respond to therapies targeting the MAPK pathway will ultimately develop resistance and undergo disease progression within 6 to 7 months of initial therapy [5].

There is evidence for a broad spectrum of genomic and non-genomic mechanisms of acquired resistance to BRAF inhibitors in metastatic melanoma. Genomic alterations thought to be responsible for resistance include activating mutations in *MEK* and *NRAS*, alternative splicing and amplification of *BRAF*, and activation of the phosphatidylinositol 3-kinase (PI3K) pathway through loss of *PTEN* or mutations in *PIK3CA* and *AKT* [7–12]. Methylome and transcriptional analysis of tumors serially biopsied prior to therapy with a MAPK pathway inhibitor and following clinical relapse suggests recurrent non-genomic mechanisms, including up-regulation of the MET receptor tyrosine kinase (RTK) and down-regulation of β-catenin-LEF1, can also be responsible for acquired resistance to these inhibitors [12].

Several studies have demonstrated an emerging role for growth factor–mediated signaling in the resistance to inhibitors targeting the MAPK pathway. Specifically, hepatocyte growth factor (HGF), the cognate ligand for the RTK MET, has been shown to convey resistance to vemurafenib and a related analog, PLX4720, in BRAF mutant melanoma cell lines [13, 14]. This resistance is driven by reactivation of the MAPK and PI3K signaling pathways. Elevated HGF levels from autocrine (tumor cell), paracrine (stromal), or systemic production were proposed to represent a novel mechanism of vemurafenib resistance. These data, along with the finding that up-regulation of MET is associated with acquired resistance to MAPK pathway inhibitor therapy suggest that combined treatment with HGF/MET inhibitors may provide additional clinical benefit.

Growth factor–mediated activation of the MAPK pathway is regulated by a complex network of extracellular signal-regulated kinase (ERK)–dependent negative feedback loops, which attenuate signal magnitude and duration. For example, MAPK pathway activation can lead to the induction of Sprouty proteins, which sequester adaptor proteins from their associated RTKs, leading to suppression of *RAS* activation and reduced downstream signaling [15, 16]. In oncogene-addicted BRAF^V600E^ mutant melanoma, flux through the MAPK pathway is high, driving robust ERK-dependent negative feedback. Feedback loops targeting RTKs and adaptor proteins would be expected to have little to no effect on MAPK pathway signaling because of their intervention upstream of activated BRAF; however, upon treatment with a BRAF inhibitor and subsequent inhibition of MAPK pathway signaling, ERK-dependent negative feedback loops are diminished, relieving significant suppression of upstream nodes and priming cells for growth factor/RTK–driven resistance. Similar resistance mechanisms have been reported in triple-negative breast cancer (TNBC) where inhibition of MAPK pathway signaling resulted in the dynamic upregulation and activation of select RTKs [17]. Combined treatment with a MEK inhibitor and pharmacologic inhibition, or small interfering RNA knockdown of the implicated RTKs, resulted in synergistic effects on TNBC cell line viability. These findings highlight a compensatory role for growth factors and their accompanying RTKs in reactivating MAPK pathway signaling and conveying resistance to downstream targeted therapy.

In this manuscript we report findings that provide further insight into the mechanism of HGF-mediated rescue of BRAF or MEK inhibition in BRAF^V600E^ mutant melanoma and demonstrate that MET and GAB1 (a key adaptor protein in HGF/MET signaling) are uniquely upregulated following MAPK pathway inhibition. The induction of MET and GAB1 primes cells for rescue by HGF, via activation of both the MAPK and PI3K signaling pathways. In addition, a strong correlation was observed between MET induction and strength of HGF rescue, suggesting that MET induction may serve as a predictive marker for identifying patients most likely to benefit from combined BRAF and MET inhibitor therapy. Finally, we demonstrate that local/tumor HGF expression may be required to convey resistance to BRAF inhibition *in vivo*, observing rescue when HGF is expressed in the tumor microenvironment but not when expressed systemically. In summary, these findings add significant and novel mechanistic insight into the potential role of HGF/MET signaling in mediating resistance to BRAF and MEK inhibitors and support the clinical evaluation of MET kinase inhibitors and HGF-neutralizing antibodies in melanoma.

## RESULTS

### HGF rescues BRAF and NRAS mutant melanoma cell lines from the growth inhibitory effects of MAPK pathway inhibition

**Figure 1:**
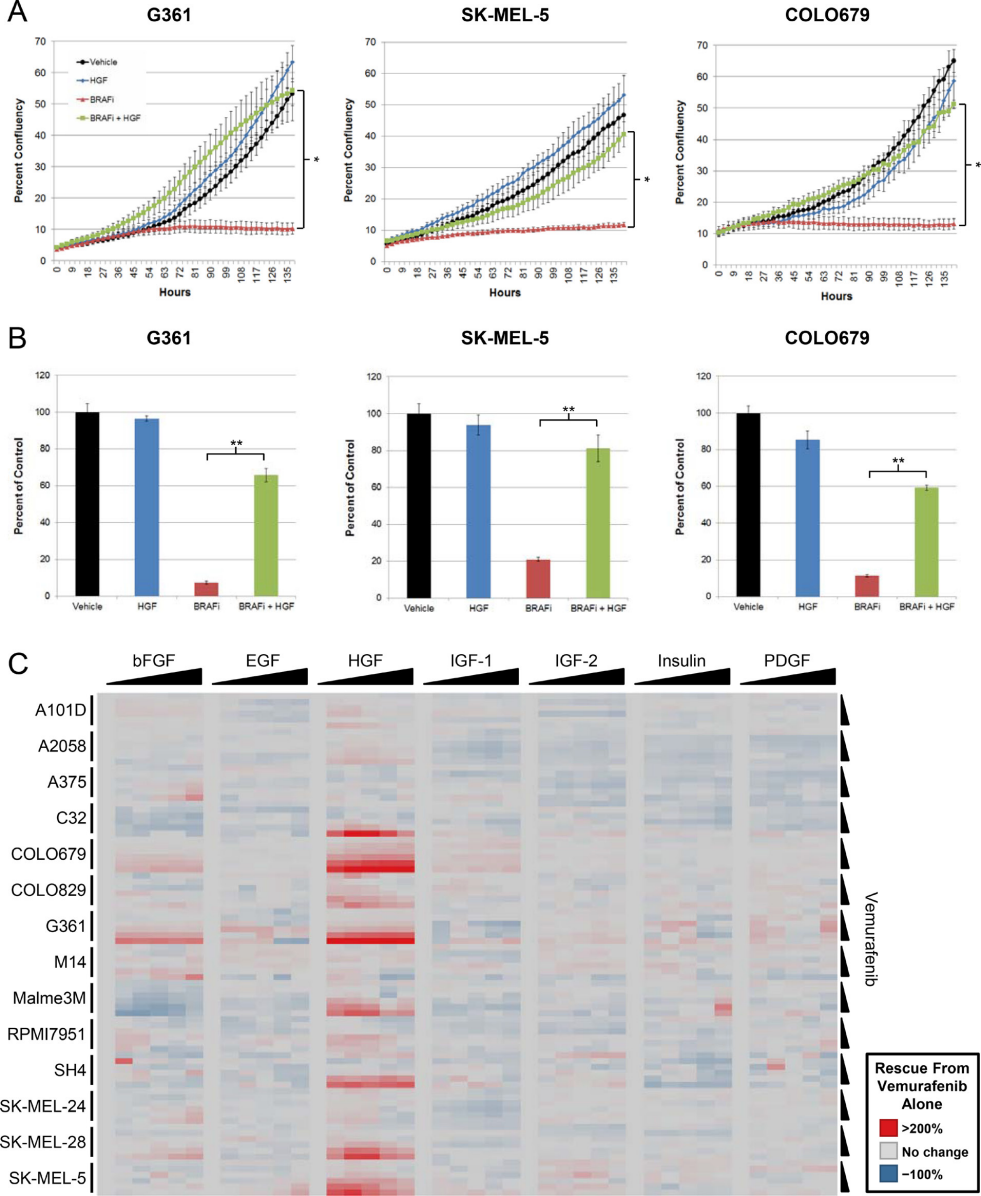
HGF treatment rescues BRAFV600E mutant melanoma cells from the effects of vemurafenib (**A**) BRAF^V600E^ mutant melanoma cell lines were treated with vehicle, vemurafenib (2 μM), HGF (100 ng/mL), or vemurafenib + HGF. Cells were imaged every 3 hours, tracking changes in confluency as a measure of proliferation. *P < 0.01. (**B**) Bar graphs depict results from terminal viability assays (ATP concentration) normalized to vehicle-treated control. Error bars represent SD across replicates (*n* = 4). **P < 0.001. (**C**) BRAFV600E mutant melanoma cell lines were treated with a serial dilution matrix of vemurafenib (3 μM top dose with five-step 1:3 serial dilution) and one of seven growth factors (300 ng/mL top dose with five-step 1:3 serial dilution; top doses of 900 and 1000 ng/mL were used for G361 and COLO679, respectively) for 72 hours. Viability was quantified and reported as percentage rescue from vemurafenib treatment alone.

To assess whether HGF could rescue BRAF^V600E^ mutant melanoma cells from BRAF inhibition, three cell lines were treated with vemurafenib in the presence or absence of HGF. Proliferative capacity was measured with live cell imaging by tracking changes in confluency over time. In agreement with its documented antiproliferative effects [18, 19], vemurafenib treatment resulted in little to no increase in confluency over the experimental duration (Figure 1A). In contrast, coadministration of HGF with vemurafenib resulted in a rapid and profound rescue of cell proliferation, achieving growth rates similar to those observed with vehicle or HGF treatment alone (Figure 1A). Terminal viability analysis identified a similar HGF-mediated rescue, with fourfold to ninefold improvements in viability over vemurafenib treatment alone (Figure 1B).

To determine the prevalence of HGF rescue, 14 BRAF^V600E^ mutant melanoma cell lines were treated with a dose titration matrix of vemurafenib and HGF (Supplementary Figure 1A). Compared with vemurafenib treatment alone (3 μM), cotreatment with HGF improved viability by > 20% in 10 cell lines, >100% in seven cell lines, and > 200% in three cell lines (Figure 1C), suggesting that HGF may represent a robust and frequent resistance mechanism to BRAF inhibitor therapy in BRAF^V600E^ mutant melanoma. In cell lines exhibiting HGF-mediated rescue, resistance was observed across a wide range of vemurafenib concentrations and with as little as 4 ng/mL HGF (Figure 1C).

To understand whether rescue was unique to HGF or shared by other RTK ligands, six additional growth factors (basic fibroblast growth factor [bFGF], epidermal growth factor [EGF], insulin-like growth factor 1 and 2 [IGF1 and IGF2], insulin, and platelet-derived growth factor [PDGF]) were tested. HGF was the predominant growth factor capable of rescuing BRAF^V600E^ mutant melanoma cell lines from vemurafenib. bFGF was the only additional growth factor to exhibited significant rescue; however, the magnitude and prevalence was far less than that observed with HGF (Figure 1C; Supplementary Figures 1B–1C).

Having observed significant HGF rescue in the initial panel of cell lines, an additional 12 patient-derived BRAF^V600E^ mutant melanoma cell lines with accompanying clinical data (Supplementary Table 1) were profiled for HGF rescue. Here we tested the ability of HGF to rescue cells from dabrafenib, a selective BRAF inhibitor, approved for the treatment of BRAF^V600E^ mutant melanoma, with similar clinical efficacy as vemurafenib but a differentiated safety profile. Three of 12 cell lines were rescued from dabrafenib treatment by > 20% (Supplementary Figure 2A), including lines derived from patient biopsies obtained before targeted therapy and lines derived from biopsies at progression after treatment with vemurafenib (Supplementary Table 1).

**Figure 2:**
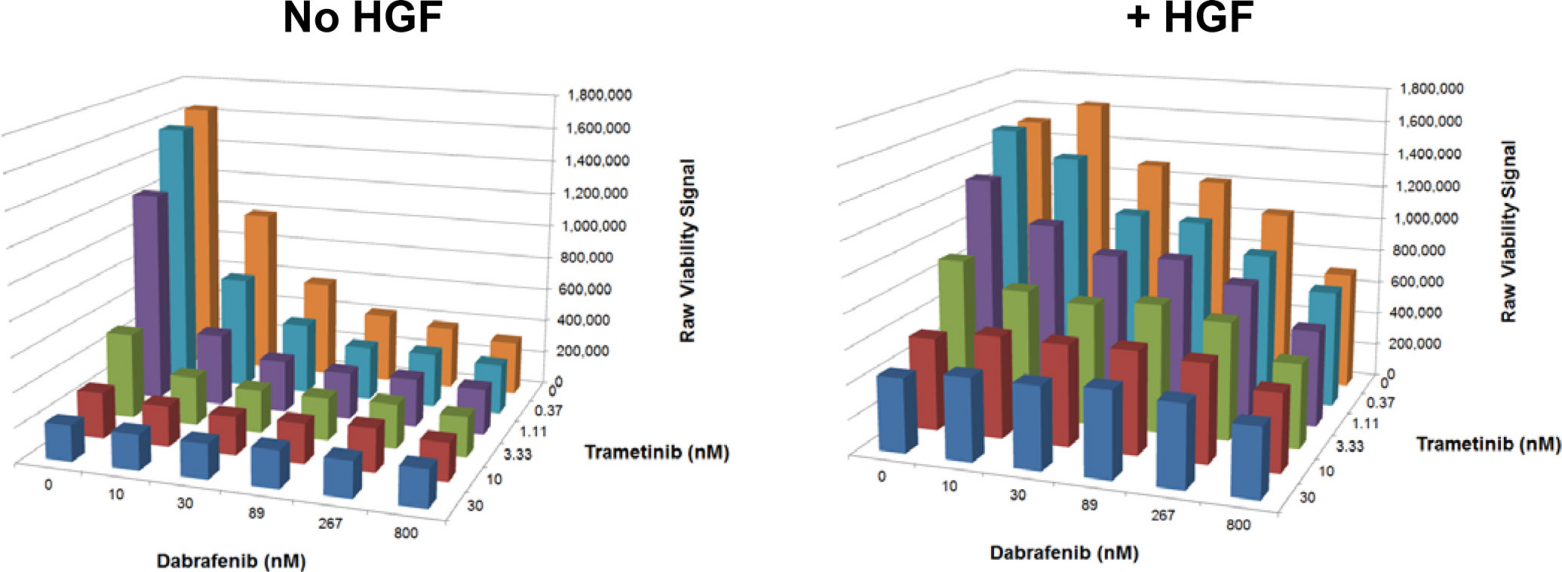
HGF treatment rescues a BRAF^V600E^ mutant melanoma cell line from the effects of a BRAF + MEK inhibitor combination G361 cells were treated with a serial dilution matrix of dabrafenib and trametinib ± HGF (25 ng/mL) for 72 hours. Viability was quantified (ATP concentration) and reported as raw luminescence (representative results from one of two independent experiments).

Because BRAF + MEK inhibitor combination therapy is replacing BRAF inhibitor monotherapy and emerging as the standard targeted therapy in BRAF^V600E^ mutant melanoma, [20–22] we sought to test whether HGF could rescue BRAF^V600E^ mutant G361 cells from treatment with dabrafenib and trametinib (selective BRAF and MEK inhibitors approved for use in combination to treat metastatic BRAF^V600E^ mutant melanoma). HGF partially rescued G361 cells from combination treatment across a wide range of concentrations (Figure 2), suggesting HGF may also confer resistance to this therapeutic combination.

In addition to *BRAF* mutations, metastatic melanoma frequently harbors activating *NRAS* mutations (15%–28%) [23–25] for which MEK inhibitor therapy has shown early clinical activity [25, 26]. To characterize whether HGF could rescue *NRAS* mutant melanoma from trametinib, six patient-derived NRAS mutant melanoma cell lines (Supplementary Table 1) were tested. Four lines were rescued by > 20% (Supplementary Figure 2B), highlighting the breadth of this rescue mechanism outside the BRAF^V600E^ mutant setting.

### Inhibition of MET signaling attenuates HGF–mediated rescue of MAPK pathway inhibition in a BRAF mutant melanoma cell Line

**Figure 3:**
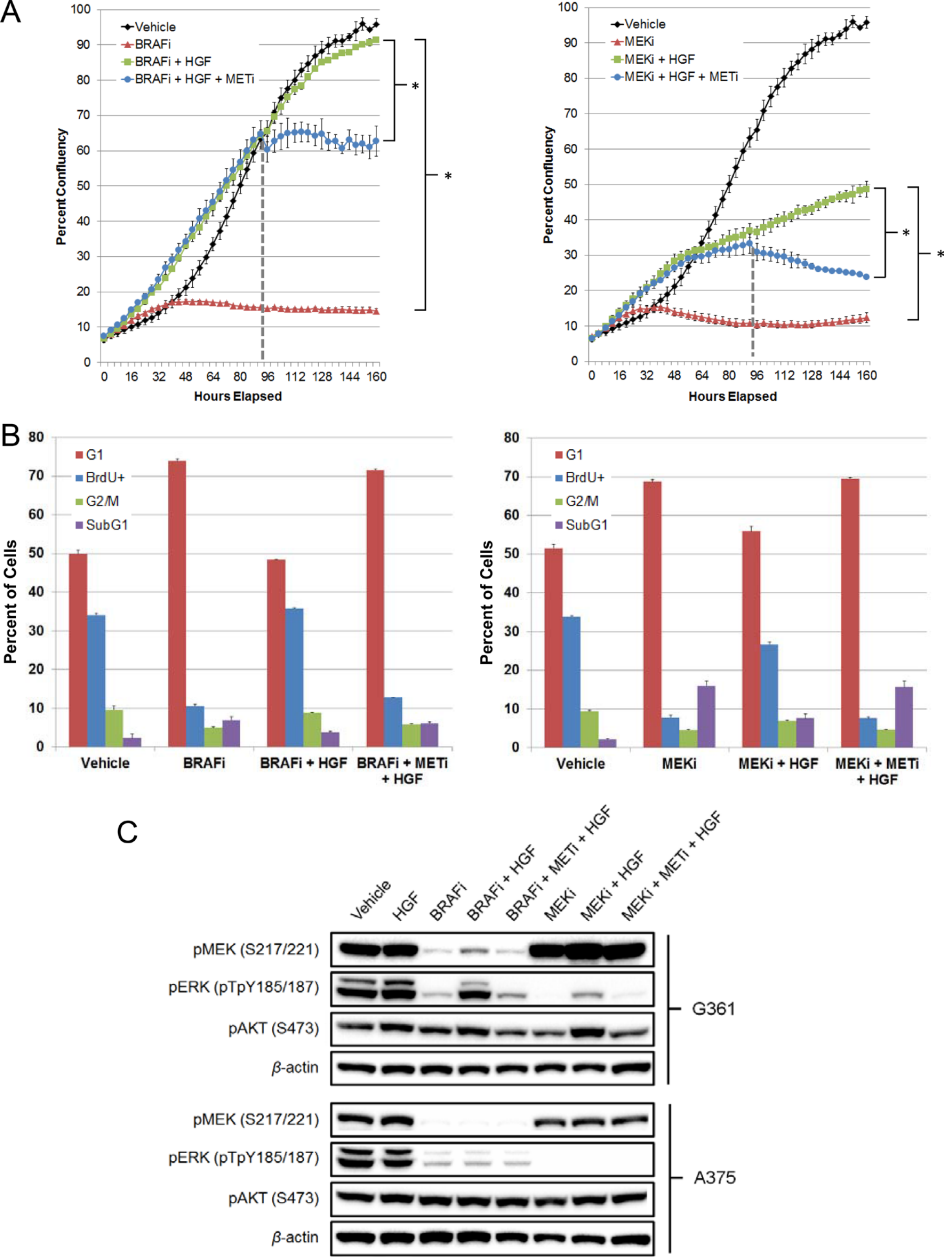
MET inhibition attenuates HGF rescue of BRAF and MEK inhibitors in G361 BRAF^V600E^ mutant melanoma cells (**A**) Cells were treated with vehicle, vemurafenib (2 μM), PD0325901 (200 nM), vemurafenib + HGF (100 ng/mL) or PD0325901 + HGF. Compound 20 (100 nM) was added at 94 hours (broken line). Cells were imaged every 4 hours, tracking changes in confluency as a measure of proliferation. Bars represent SD across replicate wells (*n* = 3). *P < 0.01. (**B**) Cells were treated with vehicle, vemurafenib (2 μM), PD0325901 (200 nM), vemurafenib + HGF (100 ng/mL), PD0325901 + HGF, vemurafenib + HGF + Compound 20 (100 nM) or PD0325901 + HGF + Compound 20 for 48 hours. BrdU labeling reagent was added for the final 2 hours of treatment. Cells were harvested, fixed, permeabilized, stained, and analyzed by flow cytometry. (**C**) HGF-rescued G361 cells and nonrescued A375 cells were treated with vehicle, HGF (100 ng/mL), vemurafenib (2 μM), PD0325901 (200 nM), Compound 20 (100 nM), or combinations for 24 hours. MAPK and PI3K signaling effects were determined by immunoblotting for pMEK (S217/221), pERK (pTpY185/187), and pAKT (S473).

MET is the cognate RTK for HGF. To confirm its role in mediating HGF rescue, Compound 20, a potent and highly selective class I MET inhibitor [27, 28], was used to treat G361 cells rescued from vemurafenib or PD0325901, a selective MEK inhibitor [29]. Compound 20 addition attenuated HGF-mediated rescue of vemurafenib, resulting in a growth curve plateau, consistent with MET inhibition and restoration of the vemurafenib antiproliferative effect (Figure 3A). To further explore the underlying mechanism of HGF rescue, we performed cell cycle analysis and confirmed that selective MET inhibition could attenuate HGF rescue by inducing a G1 growth arrest, as was seen with vemurafenib treatment alone (Figure 3B, Supplementary Figure 3A). In contrast, Compound 20 addition to cells rescued from PD0325901 produced a growth curve with a negative slope (Figure 3A), suggesting that combined MEK + MET inhibition, in the context of HGF rescue, induces greater cell-killing effects than BRAF + MET inhibition. Subsequent cell cycle analysis confirmed these findings, demonstrating that MET inhibition prevented HGF rescue from PD0325901 because of an increase in the percentage of subG1 cells and a reduction in proliferating cells (BrdU+; Figure 3B, Supplementary Figure 3B). HGF also rescued G361 cells from higher concentrations of PD0325901 (1 μM; Supplementary Figure 3C). Here, cell cycle analysis revealed robust cell killing (subG1) with PD0325901 alone; HGF addition rescued a significant portion of the subG1 cells, returning them to a proliferative state (BrdU+). Again, selective MET inhibition with Compound 20 reversed HGF-mediated rescue.

To investigate the underlying signaling changes responsible for HGF rescue, cell lysates from G361 (rescued) and A375 (not rescued) cell lines were profiled by immunoblot analysis to characterize effects on the downstream PI3K and MAPK signaling pathways when rescued from BRAF or MEK inhibition. As expected, given the presence of activating BRAF mutations, both cell lines exhibited robust pMEK and pERK levels, which were inhibited by vemurafenib treatment (Figure 3C). Cotreatment with HGF rescued a significant portion of the pMEK and pERK signals in G361 but not A375 cells. Furthermore, a similar rescue of pERK, although to a lesser extent, was observed in G361 cells upon HGF addition to PD0325901. As anticipated, MET inhibition with Compound 20 reversed the HGF rescue of MAPK pathway signaling, returning pERK and pMEK signals to levels seen with BRAF or MEK inhibitor treatment alone. HGF treatment also exhibited evidence of PI3K pathway activation, as measured by increased pAKT levels in G361 but not A375 cells (Figure 3C). To confirm the role of PI3K pathway signaling in mediating HGF rescue, AMG 511, a selective class I PI3K inhibitor [30], was added to G361 cells rescued from vemurafenib or PD0325901. In both cases, PI3K inhibition attenuated the HGF-mediated rescue of MAPK pathway inhibition (Supplementary Figure 4). These findings demonstrate that HGF rescue from BRAF or MEK inhibitor treatment was mediated by both the PI3K and MAPK signaling pathways, and selective MET inhibition blocked this underlying mechanism.

### MAPK pathway inhibition induces marked increases in MET and GAB1 transcript and protein levels, priming signaling for HGF rescue

**Figure 4:**
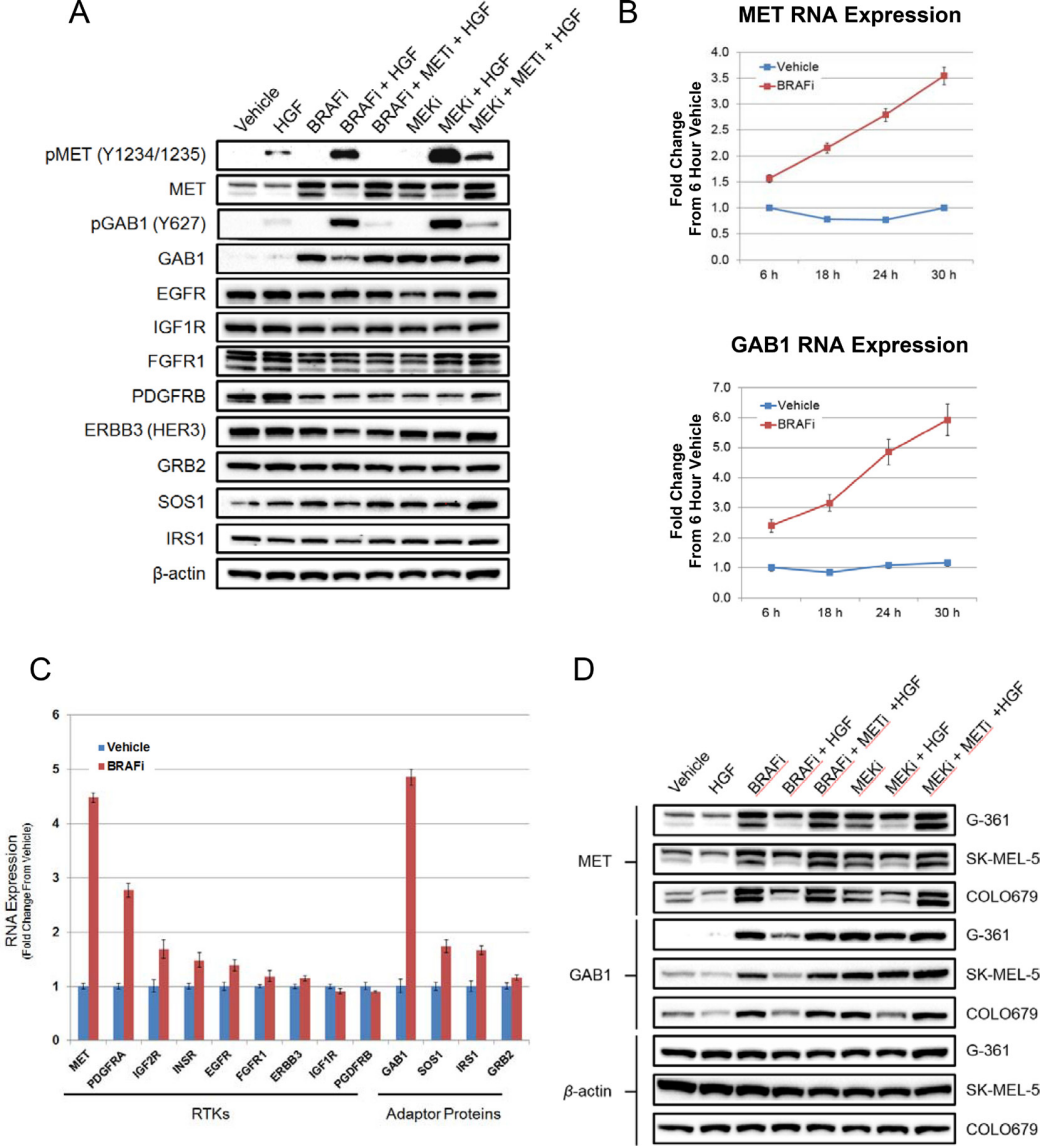
MAPK pathway inhibition mediates robust induction of MET and GAB1 levels, priming BRAF^V600E^ mutant melanoma cells for HGF-mediated rescue (**A**) Immunoblot analysis of indicated signaling proteins in BRAF^V600E^ mutant G361 cells treated with vehicle, HGF (100 ng/mL), vemurafenib (2 μM), PD0325901 (200 nM), Compound 20 (100 nM), or specified combinations for 24 hours. (**B**) RT-PCR analysis of MET and GAB1 RNA levels in G361 cells following a time course vehicle or vemurafenib (2 μM) treatment. Bars represent SD across replicates (*n* = 4). (**C**) RT-PCR analysis of RTK and adaptor protein RNA levels in G361 cells following 24-hour vehicle or vemurafenib (2 μM) treatment. Bars represent SD across replicates (*n* = 3). (**D**) Immunoblot analysis of MET and GAB1 levels in BRAF^V600E^ mutant melanoma cell lines rescued from the effects of BRAF inhibition by HGF. Cells were treated as in 4A.

Having characterized the effects of HGF rescue on downstream PI3K and MAPK signaling pathways, we next investigated the role of MET and GAB1, a key adaptor protein in HGF/MET signaling [12, 31], in mediating resistance. HGF treatment of G361 cells resulted in a limited increase in pMET and pGAB1 levels (Figure 4A). In vemurafenib- or PD0325901-treated cells, HGF induced far greater increases in the phosphorylation state of these two proteins. Immunoblot analysis of total MET revealed two bands, both of which were induced following treatment with either a BRAF or MEK inhibitor (Figure 4A). The higher molecular weight band (~170 kDa) represents the MET proreceptor (p170met), which is cleaved to form the mature receptor composed of disulfide-linked alpha and beta subunits [32]. The lower molecular weight band corresponds to the beta subunit (~140 kDa), the levels of which were inversely correlated with MAPK pathway signaling (pERK) (Figures 3C and 4A), suggesting that MAPK pathway inhibition may promote proteolytic cleavage of the MET proreceptor to its mature alpha and beta subunits. Furthermore, a significant increase in total GAB1 expression was similarly observed upon treatment with either a BRAF or MEK inhibitor (Figure 4A). Taken together these data suggest that the increases in total MET and GAB1 may prime BRAF mutant melanoma cells for HGF-mediated rescue. As expected, cotreatment with Compound 20 significantly reduced MET and GAB1 phosphorylation levels. To understand whether this induction in total protein following MAPK pathway inhibition was unique to MET and GAB1, we profiled the same G361 lysates with antibodies to a panel of RTKs and adaptor proteins, some of which are known targets of MAPK pathway–mediated negative feedback loops [33]. Only MET and GAB1 exhibited significant induction following vemurafenib or PD0325901treatment (Figure 4A), indicating this induction may contribute to the underlying mechanism of HGF rescue and explain the unique ability of HGF to rescue BRAF mutant melanoma cells from MAPK pathway inhibition.

Previous reports have documented a network of ERK-dependent negative feedback loops that attenuate MAPK signaling, some of which regulate signaling at the RTK level [15, 33, 34]. In BRAF^V600E^ mutant melanoma, where robust MAPK pathway signaling is present, we hypothesized that MET and GAB1 levels were repressed by an ERK-dependent negative feedback mechanism. Upon treatment with a BRAF or MEK inhibitor, ERK-dependent feedback would be expected to diminish, resulting in elevated MET and GAB1. To further characterize the increase in MET and GAB1 after vemurafenib treatment, we performed a time course experiment in G361 cells (Supplementary Figure 5). Although ERK phosphorylation was inhibited within 15 minutes of treatment, increases in MET and GAB1 were not observed until 8 hours post-treatment. This delay suggested changes in transcription may be regulating the protein level increases. To test this hypothesis, qRT-PCR was used to evaluate changes in *MET* and *GAB1* transcript levels. A robust increase in *MET* and *GAB1* transcripts following a time course of vemurafenib treatment was observed, reaching a maximal induction of 3.5-fold and 6-fold, respectively, at 30 hours (Figure 4B). Further characterization revealed the increases in *MET* and *GAB1* transcripts were unique among a panel of tested RTK and adaptor proteins (Figure 4C). The only other transcript to exhibit greater than twofold increase following vemurafenib treatment was PDGFRA; however, no PDGFRA protein was detected in G361 cells in the basal or MAPK pathway inhibited state (unpublished data). To confirm this observation was not limited to G361 cells, two additional HGF-rescuable BRAF^V600E^ mutant melanoma cell lines, SK-MEL-5 and COLO679, were characterized for MET and GAB1 induction. Both lines exhibited clear increases in MET and GAB1 following vemurafenib and PD0325901 treatment (Figure 4D).

### Induction of MET following vemurafenib treatment may serve as a predictive biomarker for identifying patients best suited for MET inhibitor combination therapy

**Figure 5:**
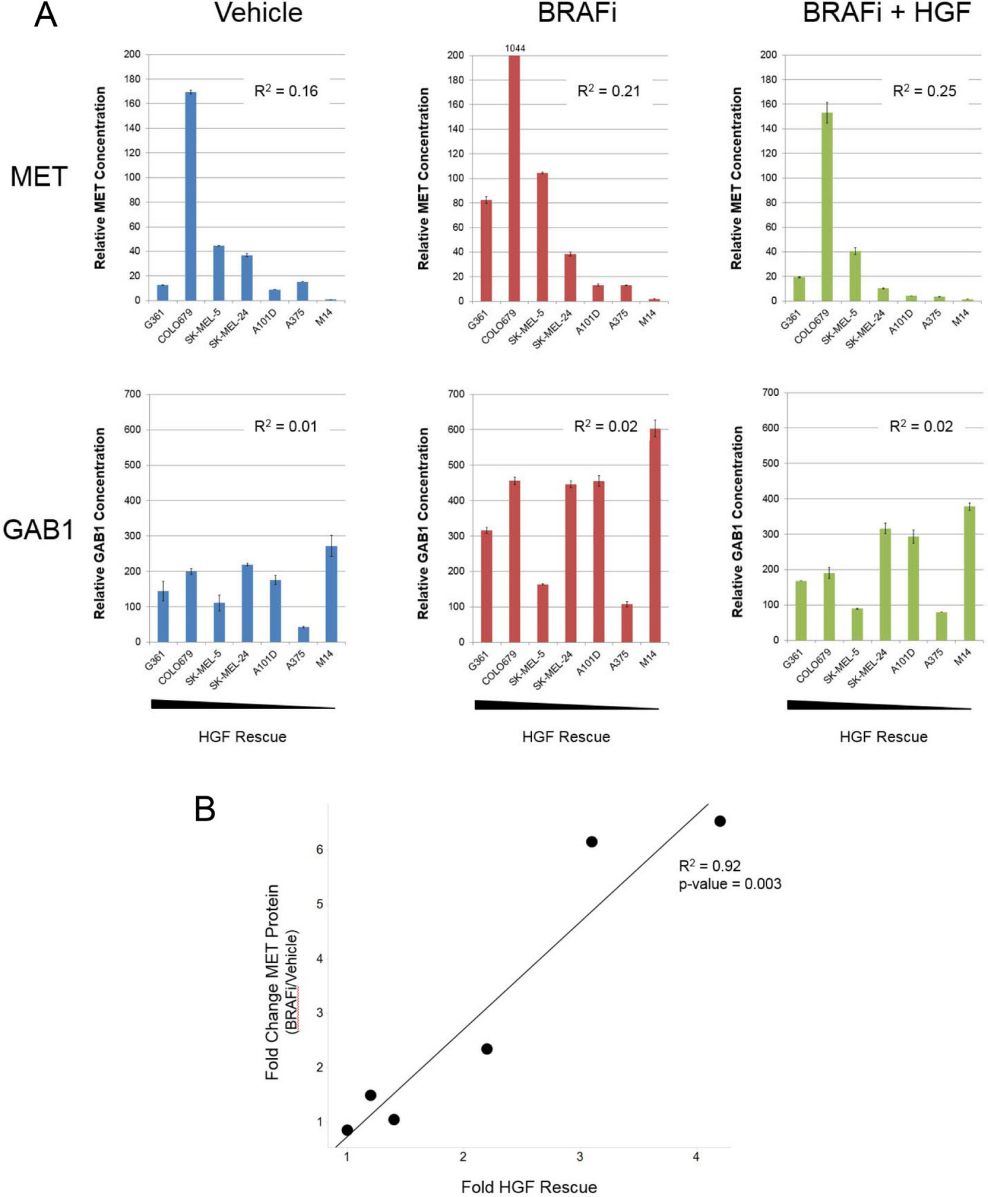
Fold increase in MET following BRAFi treatment predicts for strength of HGF rescue in BRAF^V600E^ mutant melanoma cells (**A**) Seven BRAF^V600E^ mutant melanoma cell lines exhibiting varying degrees of HGF-mediated rescue (rank order left to right based on strength of HGF rescue) were treated with vehicle, vemurafenib (2 μM), HGF (100 ng/mL), or vemurafenib + HGF for 24 hours. MET and GAB1 protein levels were measured using MSD assays. *R*^2^ values derived from linear regression analysis of MET or GAB1 values and fold HGF rescue for individual cell lines. (**B**) Correlation between fold HGF rescue and fold change in MET following vemurafenib treatment. M14 was omitted from analysis because MET levels fell below fell below the limit of detection.

Profiling BRAF^V600E^ mutant melanoma cell lines identified considerable diversity in the degree of HGF rescue from vemurafenib (Figure 1C). Given this spectrum of response, we tested whether MET and GAB1 induction following vemurafenib treatment would predict for the degree of HGF rescue. We selected a subset of cell lines spanning a range of HGF rescue (Supplementary Table 2) and quantitatively measured total and phosphorylated MET and GAB1 in each following vehicle, vemurafenib, or vemurafenib + HGF treatment. As with previous signaling results (Figure 4D), clear induction of total MET and GAB1 was observed in several cell lines following vemurafenib treatment (Figure 5A). Although elevated upon vemurafenib treatment, GAB1 levels were not predictive of HGF rescue under any treatment conditions tested (Figure 5A). In contrast, MET levels following vemurafenib treatment demonstrated an enhanced association with HGF rescue strength. Here, the four cell lines with highest MET expression (G361, COLO679, SK-MEL-4, and SK-MEL-24) also exhibited strongest HGF rescue. Similarly, pMET and pGAB1 levels following vemurafenib and HGF cotreatment were also predictive (Supplementary Figure 6), displaying similar patterns to those for total MET following vemurafenib treatment alone. The most compelling association was observed when the HGF rescue strength was compared with the fold increase in MET following vemurafenib treatment (*R*^2^ = 0.92, *p*-value = 0.003; Figure 5B), suggesting that total MET induction may serve as a predictive biomarker for identifying patients most likely to benefit from combined therapy with MET and MAPK pathway inhibitors.

### Local/tumor HGF expression is required to convey resistance to BRAF inhibition in a BRAF^V600E^ mutant melanoma xenograft model

**Figure 6:**
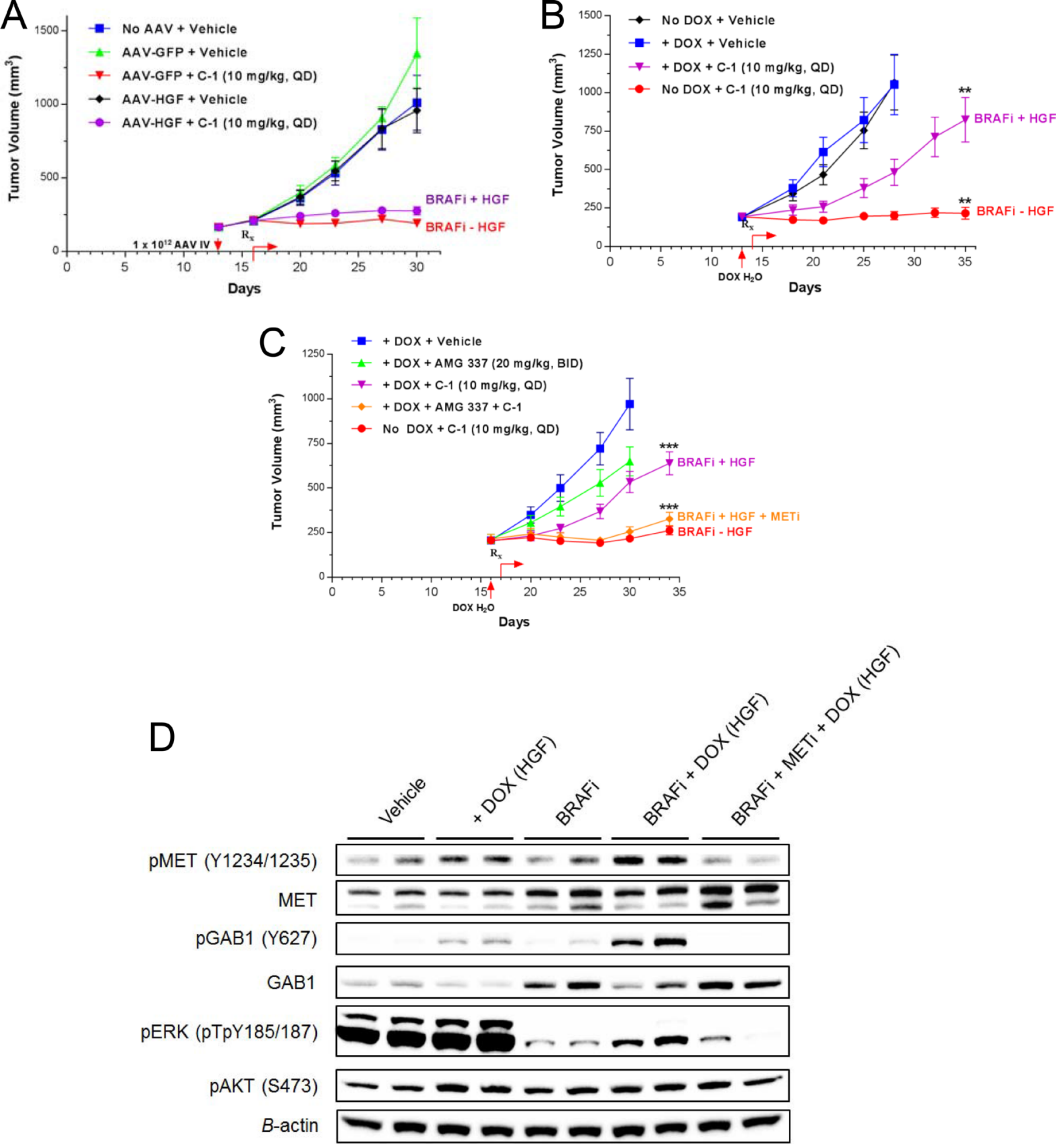
Elevated local/tumor HGF expression is required for resistance to BRAF inhibition *in vivo* (**A**) Athymic nude mice bearing BRAF^V600E^ mutant melanoma G361 tumor xenografts were treated intravenously with recombinant AAV vector containing human HGF (AAV-HGF) or GFP (AAV-GFP; 1 × 10^12^ viral particles/mouse). Three days post-administration, mice were treated with C-1 (10 mg/kg QD) or vehicle. Tumor volumes were recorded twice weekly (mean ± SEM). (**B**) Athymic nude mice bearing Tet-HGF-G361 tumor xenografts were administered doxycycline (0.1 mg/mL) to induce HGF expression. C-1 treatment was initiated the next day (10 mg/kg QD). Tumor volume was monitored as in 6A. **P < 0.001 (BRAFi + HGF versus BRAFi – HGF). (**C**)| Tet-HGF-G361 tumor xenograft studies were conducted as in 6B. Mice were treated with vehicle, C-1 (10 mg/kg QD), AMG 337 (20 mg/kg BID), or C-1 and AMG 337 in combination. ***P < 0.0001 (BRAFi + HGF versus BRAFi + HGF + METi or BRAFi – HGF). (**D**) Immunoblot analysis of signaling proteins from pharmacodynamic study of Tet-HGF-G361 tumor xenograft samples collected 6 hours after final drug treatment.

Previous reports have identified associations between tumor and plasma HGF expression and clinical response to vemurafenib [13, 14]. To investigate whether circulating HGF versus HGF present in the tumor microenvironment could mediate resistance to BRAF inhibition, G361 xenografts were used to generate models of systemic and local/tumor HGF expression (Supplementary Figure 7). To model systemic HGF expression, mice bearing G361 xenografts were treated with recombinant AAV containing an expression cassette for either human HGF (AAV-HGF) or GFP (AAV-GFP) as control. AAV-HGF treatment resulted in transduction of the liver and subsequent systemic expression of HGF. Mice were then treated with the BRAF inhibitor C-1 (10 mg/kg, QD, by mouth [PO]) or vehicle and tumor growth was monitored to characterize HGF-mediated rescue. C-1 was used in place of vemurafenib for this experiment as prior work had established a well characterized relationship between C-1 dose, exposure and target coverage in tumor xenograft models [35]. Systemic HGF expression driven via AAV-HGF at two different viral titers failed to rescue G361 xenografts from the growth inhibitory effects of BRAF inhibition following treatment with C-1 (Figure 6A, Supplementary Figure 8).

To model local/tumor HGF expression, mice bearing Tet-HGF-G361 xenografts, with or without doxycycline in the drinking water, were treated with C-1 or vehicle. Local/tumor HGF expression conveyed a significant rescue of C-1 driven tumor growth inhibition (*P* < 0.001; Figure 6B), suggesting local HGF expression may be required to mediate BRAF inhibitor resistance. Comparison of plasma and tumor HGF levels from the systemic and local/tumor HGF models revealed the highest plasma HGF levels in the high-dose AAV-HGF animals, whereas the highest tumor HGF levels were found in the Tet-HGF model (Supplementary Figure 9).

Our *in vitro* studies would predict that combined BRAF + MET inhibition should reverse the HGF-mediated rescue of the BRAF inhibitor C-1 observed in the Tet-HGF-G361 xenograft study. Furthermore, analysis of the *in-vitro* studies would indicate that complete suppression of downstream MET signaling would be necessary to reverse HGF-mediated resistance to C-1. To achieve complete inhibition of MET signaling *in-vivo*, the MET kinase inhibitor AMG 337 was dosed at 20 mg/kg BID which is predicted to achieve plasma concentrations sufficient for > 90% inhibition of MET for 24 hrs [27]. Combined treatment of the BRAF inhibitor C-1 (10 mg/kg, QD, PO) and AMG 337 (20 mg/kg, BID, PO) completely attenuated the HGF-mediated rescue of C-1 in Tet-HGF-G361 xenografts (Figure 6C). Subsequent pharmacodynamic analysis of downstream signaling pathways demonstrated HGF-dependent rescue of pERK upon the addition of doxycycline to the drinking water of C-1–treated mice (Figure 6D, Supplementary Figure 10). The increase in ERK phosphorylation was attenuated upon AMG 337 addition to C-1 treatment. As observed with BRAF inhibition *in vitro*, clear induction of total MET and GAB1 was observed following C-1 treatment. Additionally, HGF-mediated increases in the phosphorylation of these two proteins were more pronounced following cotreatment with C-1 and doxycycline compared with doxycycline alone. These results suggest that repression of ERK-mediated negative feedback loops following BRAF inhibitor treatment primes total MET and GAB1 for HGF rescue, activating downstream signaling pathways and promoting tumor growth (Supplementary Figure 11).

## DISCUSSION

Resistance remains a significant limiting factor in achieving robust and durable clinical responses to targeted cancer therapy. More specifically, resistance to small-molecule BRAF and MEK kinase inhibitors has been well-documented as limiting the efficacy of these agents in BRAF^V600E^ mutant melanoma. Resistance is attributed to multiple mechanisms, including growth factor–mediated activation of RTKs [13, 14]. In this study, we highlight the ability of HGF to rescue BRAF^V600E^ mutant melanoma cell lines from the effects of BRAF and/or MEK inhibition, observing evidence of HGF rescue in the majority of profiled cell lines. Furthermore, we extended this observation to NRAS mutant melanoma. Here HGF rescued four of six NRAS mutant melanoma cell lines from MEK inhibition.

Subsequent mechanistic studies revealed robust and rapid increases in MET and GAB1 transcript and protein levels following treatment with MAPK pathway inhibitors. This finding was consistent with studies documenting elevated MET transcript levels in biopsies from tumors resistant to BRAF/MEK inhibitors [12]. Furthermore, cotreatment with HGF and a BRAF or MEK inhibitor increased MET and GAB1 phosphorylation beyond that observed with HGF alone. Downstream increases in ERK and AKT phosphorylation were also observed, suggesting that changes in total MET and GAB1 prime BRAF mutant melanoma cells for HGF-mediated rescue via downstream activation of the PI3K and MAPK signaling pathways (Supplementary Figure 11).

Detection of HGF-mediated resistance within the first few hours of inhibitor treatment indicated that the underlying resistance mechanism was dynamic and adaptive in nature and inherently different from the well-documented acquired resistance mechanisms typically observed following extended drug treatment (weeks to months) [36]. Adaptive mechanisms are thought to represent the first step in a multistep process that culminates in therapeutic resistance. By providing the tumor cell with an immediate survival benefit, adaptive mechanisms allow cells to persist over time until a more robust and stable resistance mechanism presents, such as the acquisition of a drug-resistant genetic alteration [37]. Targeting the initial adaptive mechanism that promotes the evolution and persistence of drug tolerant cells may prevent the emergence of more stable acquired resistance and subsequently increase the duration and magnitude of therapeutic response. The unique role of HGF/MET signaling in conferring resistance to MAPK pathway inhibitors in BRAF and NRAS mutant melanoma suggests that combination therapy with small-molecule MET inhibitors may provide additional benefit beyond BRAF and MEK inhibitors alone. To test this, BRAF^V600E^ mutant melanoma cell lines, rescued from the effects of BRAF or MEK inhibitors by HGF, were cotreated with a selective MET inhibitor. Clear attenuation of HGF rescue was observed, returning cells to a growth-arrested state when combined with a BRAF inhibitor and inducing cell death when combined with a MEK inhibitor. Subsequent analysis of underlying changes in pathway signaling revealed a reversal of the HGF-mediated rescue of PI3K and MAPK pathway signaling upon MET inhibition. We also determined that the strongest measure for predicting strength of HGF rescue in the BRAF mutant melanoma cell lines was the fold increase in MET levels following BRAF inhibitor treatment, suggesting that the induction of total MET may serve as a predictive biomarker to ascertain which patients with BRAF^V600E^ mutant melanoma are most likely to exhibit adaptive resistance and benefit from MET inhibitor combination therapy. Initial reports documenting HGF-mediated resistance to BRAF inhibition in BRAF mutant melanoma identified correlations between HGF expression levels and patient response to BRAF and/or MEK inhibitor therapy. Using immunohistochemistry analysis of melanoma biopsies, an improved response to BRAF and/or MEK inhibitor therapy was found in patients with no detectable stromal HGF expression, as compared with those expressing HGF [13]. Alternatively, significant improvements in PFS and OS were found in patients with low plasma HGF levels [14]. To further investigate whether circulating HGF versus HGF present in the tumor microenvironment could mediate resistance to BRAF inhibition, we modeled systemic and local/tumor HGF expression systems in mice. We showed that systemic expression of HGF via AAV-HGF treatment failed to promote resistance to BRAF inhibition in G361 tumor xenografts, whereas local/tumor doxycycline–induced expression of HGF was capable of conferring resistance. These results suggest that HGF expression in the local/tumor microenvironment may be required to successfully promote resistance to BRAF inhibition.

Furthermore, treatment with AMG 337 (a selective MET inhibitor) completely attenuated HGF-mediated rescue. However, the reversal of HGF-mediated resistance to BRAF inhibition required the administration of AMG 337 at a dose and schedule that would provide greater than 90% target coverage for 24hrs. This observation has potential translational significance, suggesting that circumvention of HGF mediated resistance to therapies targeting the MAPK pathway will require treatment with therapeutics capable of achieving complete inhibition of MET signaling.

These findings demonstrate the role of HGF/MET signaling in mediating resistance to BRAF and MEK inhibitors in BRAF and NRAS mutant melanoma. Monitoring changes in total MET and tumor HGF levels may have clinical utility for identifying patients most likely to benefit from combination therapy with inhibitors targeting the MAPK pathway and HGF/MET signaling.

## MATERIAS AND METHODS

### Cell lines and reagents

BRAF and NRAS mutant melanoma cell lines were obtained from various sources (Supplementary Methods). The tetracycline (Tet)-HGF-G361 cell line was generated as described (Supplementary Methods). Cell lines were maintained in vendor- or investigator-recommended media. Vemurafenib was purchased from Jubilant Life Sciences; the BRAF inhibitor C-1 [35, 38], dabrafenib, trametinib, the MET inhibitors AMG 337 and Compound 20 (an AMG 337 analog), the PI3K inhibitor AMG 511, and the MEK inhibitor PD0325901 were supplied by Amgen Inc. Antibodies against pMEK (S217/221), pAKT (S473), pMET (Y1234/1235), pGAB1 (Y627), MET, GRB2, SOS1, ERBB3, EGFR, IGF1R, FGFR1, IRS1, and PDGFRB were obtained from Cell Signaling Technology. Antibodies against pERK (T185/Y187; Life Technologies), GAB1 (Millipore), and beta-actin (Abcam) were also obtained.

### Flow cytometry

Cell surface expression of MET was measured by flow cytometry as described in Supplementary Methods.

### Animals

Female 4-6-week-old athymic nude mice (Charles River Laboratories) were housed according to all Association for Assessment and Accreditation of Laboratory Animal Care specifications. Experimental procedures were performed in accordance with Institutional Animal Care and Use Committee and US Department of Agriculture regulations.

### Adeno-associated virus protein expression constructs

Recombinant adeno-associated virus (AAV) vectors containing expression cassettes for human HGF (AAV-HGF) or green fluorescent protein (AAV-GFP) were produced and purified as previously described [39].

### Live content imaging analysis and cell viability assay

Cells were seeded in 96-well plates at optimal density. Following overnight incubation, cells were treated with compounds and/or growth factors at specified concentrations. Plates were imaged at 20× magnification every 3 hours for 5 to 8 days using an IncuCyte Live-Cell Imaging System (Essen BioSciences) to monitor confluency changes over time. After imaging, cell viability was assessed using CellTiter-Glo^®^ Luminescent Cell Viability Assay kits (Promega).

### Cell cycle analysis

Cells were seeded in six-well plates. After overnight incubation, cells were treated with compounds and/or growth factors for 48 hours. Bromodeoxyuridine (BrdU) labeling reagent was added for the final 2 hours. Cells were harvested, fixed in 90% methanol at −20°C, then treated with 2N HCl and 0.5% Triton X-100, and stained with anti-BrdU Alexa-647 and propidium iodide. Finally, cells were RNase-treated and analyzed via an LSRII flow cytometer (BD Biosciences), counting 20,000 events/treatment condition.

### Immunoblot analysis

Treated cells and xenograft tumors were lysed in radioimmunoprecipitation assay (RIPA) lysis buffer containing phosphatase and protease inhibitors. Lysates were cleared by centrifugation and concentrations were determined by Bio-Rad protein assay. Lysates were resolved using NuPAGE^®^ gels (Life Technologies); membranes were blotted using total or phospho-specific antibodies (Supplementary Methods).

### Electro-chemiluminescence immuno assay

Lysates were prepared as described for immunoblot analysis and protein levels profiled using Meso Scale Discovery (MSD) 96-well singleplex assays. The standard manufacturer protocol was used for each commercially produced assay (total and pMET [Y1349], pERK1/2 [T185/Y187], and pAKT [S473]). Custom GAB1 printed plates were developed using a total GAB1 capture antibody (Santa Cruz Biotechnology). Primary detection antibodies (2 μg/mL) were used for total GAB1 (Bethyl Laboratories) and p-GAB1 (Y627; Cell Signaling Technology); a commercial MSD SULFO-TAG labeled goat-anti-rabbit secondary detection antibody (2 μg/mL) was used. The standard manufacturer-recommended MSD protocol was used to detect total GAB1; the protocol was modified for phospho-GAB1 to include overnight incubation at 4°C and a 2-hour room temperature incubation with Y627 antibody. See Supplementary Methods for additional details.

### Quantitative real-time polymerase chain reaction

Cellular RNA was extracted using QIAshredder and RNeasy Mini kits (Qiagen). Transcript levels were assayed using the TaqMan^®^ One-Step RT-PCR Master Mix Reagents Kit and TaqMan^®^ Gene Expression Assays (ThermoFisher Scientific). Quantitative real-time polymerase chain reaction (qRT-PCR) reactions were run as three or four technical replicates and assayed using the Prism^®^ 7900HT (Applied Biosystems), applying the relative quantification (ΔΔCt) method. Data were analyzed with SDS2.3, RQ Manager v1.2, and Data Assist v3.01 software (Applied Biosystems), using glyceraldehyde 3-phosphase dehydrogenase as the endogenous control.

### Growth factor rescue experiments

BRAF and NRAS mutant melanoma cell lines were treated with vemurafenib, dabrafenib, trametinib, or PD0325901 and HGF or specified growth factors. Cells were incubated for 72 hours; viability was assessed using CellTiter-Glo Luminescent Cell Viability Assay kits. Luminescence was measured by EnVision^®^ plate reader (PerkinElmer). Rescue magnitude was reported as percentage difference in raw luminescence (drug treatment + indicated growth factor versus drug treatment alone).

### Xenograft studies

In the systemic model, mice were injected subcutaneously with 5 × 10^6^ G361 cells. When the tumor volume was approximately 200 mm^3^, mice were randomly assigned to receive intravenous injections of AAV-HGF or AAV-GFP (*n* = 10 each; 1–5 × 10^12^ viral particles/mouse in 0.2 mL phosphate-buffered saline [PBS], single dose). Inhibitor treatment began 3 days after AAV administration. In the tumor/autocrine model, mice received 5 × 10^6^ G361cells engineered to express HGF under the control of a tetracycline-inducible promoter (Tet-HGF). Drinking water containing 0.1 mg/mL doxycycline was administered at randomization to induce tumor HGF expression, and inhibitor treatment began 1 day later. Mice were treated orally with vehicle, C-1 (10 mg/kg once daily [QD]), AMG 337 (20 mg/kg twice daily [BID]), or C-1 and AMG 337 in combination. Tumor volumes and body weights were measured twice weekly. A pharmacodynamic study was performed on mice harboring Tet-HGF-G361 xenografts. Mice received 24 hours of dosing; tumors were harvested 6 hours after the 24-hour dose (second dose of C-1, third dose of AMG 337).

### Determination of plasma and tumor hepatocyte growth factor levels

Human HGF levels were measured in plasma and tumor lysates from mice bearing G361 xenografts using the MSD 96-well singleplex HGF assay. To quantify plasma HGF levels, blood was collected via cardiac puncture into ethylenediaminetetraacetic acid-coated tubes; plasma was separated by centrifugation and processed in the HGF assay according to the manufacturer's protocol (25 μL plasma/well). 50 μg lysate/well were used to measure tumor HGF levels.

### Statistical analysis

For *in vitro* analyses, error bars represent the standard deviation (SD) from replicate data points; statistical significance was established using a two-tailed unpaired *t* test assuming unequal variance. Statistical significance from xenograft studies was determined by repeated measures analysis of variance.

## SUPPLEMENTARY MATERIALS FIGURES AND TABLES





## References

[1] Davies H, Bignell GR, Cox C, Stephens P, Edkins S, Clegg S, Teague J, Woffendin H, Garnett MJ, Bottomley W, Davis N, Dicks E, Ewing R (2002). Mutations of the BRAF gene in human cancer. Nature.

[2] Wan PT, Garnett MJ, Roe SM, Lee S, Niculescu-Duvaz D, Good VM, Jones CM, Marshall CJ, Springer CJ, Barford D, Marais R, Cancer Genome P (2004). Mechanism of activation of the RAF-ERK signaling pathway by oncogenic mutations of B-RAF. Cell.

[3] Guan J, Gupta R, Filipp FV (2015). Cancer systems biology of TCGA SKCM: efficient detection of genomic drivers in melanoma. Sci Rep.

[4] Chapman PB, Hauschild A, Robert C, Haanen JB, Ascierto P, Larkin J, Dummer R, Garbe C, Testori A, Maio M, Hogg D, Lorigan P, Lebbe C (2011). Improved survival with vemurafenib in melanoma with BRAF V600E mutation. N Engl J Med.

[5] McArthur GA, Chapman PB, Robert C, Larkin J, Haanen JB, Dummer R, Ribas A, Hogg D, Hamid O, Ascierto PA, Garbe C, Testori A, Maio M (2014). Safety and efficacy of vemurafenib in BRAF(V600E) and BRAF(V600K) mutation-positive melanoma (BRIM-3): extended follow-up of a phase 3, randomised, open-label study. Lancet Oncol.

[6] Merlino G, Herlyn M, Fisher DE, Bastian BC, Flaherty KT, Davies MA, Wargo JA, Curiel-Lewandrowski C, Weber MJ, Leachman SA, Soengas MS, McMahon M, Harbour JW (2016). The state of melanoma: challenges and opportunities. Pigment Cell Melanoma Res.

[7] Van Allen EM, Wagle N, Sucker A, Treacy DJ, Johannessen CM, Goetz EM, Place CS, Taylor-Weiner A, Whittaker S, Kryukov GV, Hodis E, Rosenberg M, McKenna A (2014). The genetic landscape of clinical resistance to RAF inhibition in metastatic melanoma. Cancer Discov.

[8] Trunzer K, Pavlick AC, Schuchter L, Gonzalez R, McArthur GA, Hutson TE, Moschos SJ, Flaherty KT, Kim KB, Weber JS, Hersey P, Long GV, Lawrence D (2013). Pharmacodynamic effects and mechanisms of resistance to vemurafenib in patients with metastatic melanoma. J Clin Oncol.

[9] Shi H, Hugo W, Kong X, Hong A, Koya RC, Moriceau G, Chodon T, Guo R, Johnson DB, Dahlman KB, Kelley MC, Kefford RF, Chmielowski B (2014). Acquired resistance and clonal evolution in melanoma during BRAF inhibitor therapy. Cancer Discov.

[10] Poulikakos PI, Persaud Y, Janakiraman M, Kong X, Ng C, Moriceau G, Shi H, Atefi M, Titz B, Gabay MT, Salton M, Dahlman KB, Tadi M (2011). RAF inhibitor resistance is mediated by dimerization of aberrantly spliced BRAF(V600E). Nature.

[11] Rizos H, Menzies AM, Pupo GM, Carlino MS, Fung C, Hyman J, Haydu LE, Mijatov B, Becker TM, Boyd SC, Howle J, Saw R, Thompson JF (2014). BRAF inhibitor resistance mechanisms in metastatic melanoma: spectrum and clinical impact. Clin Cancer Res.

[12] Hugo W, Shi H, Sun L, Piva M, Song C, Kong X, Moriceau G, Hong A, Dahlman KB, Johnson DB, Sosman JA, Ribas A, Lo RS (2015). Non-genomic and Immune Evolution of Melanoma Acquiring MAPKi Resistance. Cell.

[13] Straussman R, Morikawa T, Shee K, Barzily-Rokni M, Qian ZR, Du J, Davis A, Mongare MM, Gould J, Frederick DT, Cooper ZA, Chapman PB, Solit DB (2012). Tumour micro-environment elicits innate resistance to RAF inhibitors through HGF secretion. Nature.

[14] Wilson TR, Fridlyand J, Yan Y, Penuel E, Burton L, Chan E, Peng J, Lin E, Wang Y, Sosman J, Ribas A, Li J, Moffat J (2012). Widespread potential for growth-factor-driven resistance to anticancer kinase inhibitors. Nature.

[15] Hanafusa H, Torii S, Yasunaga T, Nishida E (2002). Sprouty1 and Sprouty2 provide a control mechanism for the Ras/MAPK signalling pathway. Nat Cell Biol.

[16] Casci T, Vinos J, Freeman M (1999). Sprouty, an intracellular inhibitor of Ras signaling. Cell.

[17] Duncan JS, Whittle MC, Nakamura K, Abell AN, Midland AA, Zawistowski JS, Johnson NL, Granger DA, Jordan NV, Darr DB, Usary J, Kuan PF, Smalley DM (2012). Dynamic reprogramming of the kinome in response to targeted MEK inhibition in triple-negative breast cancer. Cell.

[18] Yang H, Higgins B, Kolinsky K, Packman K, Go Z, Iyer R, Kolis S, Zhao S, Lee R, Grippo JF, Schostack K, Simcox ME, Heimbrook D (2010). RG7204 (PLX4032), a selective BRAFV600E inhibitor, displays potent antitumor activity in preclinical melanoma models. Cancer Res.

[19] Joseph EW, Pratilas CA, Poulikakos PI, Tadi M, Wang W, Taylor BS, Halilovic E, Persaud Y, Xing F, Viale A, Tsai J, Chapman PB, Bollag G (2010). The RAF inhibitor PLX4032 inhibits ERK signaling and tumor cell proliferation in a V600E BRAF-selective manner. Proc Natl Acad Sci U S A.

[20] Menzies AM, Long GV (2014). Dabrafenib and trametinib, alone and in combination for BRAF-mutant metastatic melanoma. Clin Cancer Res.

[21] Long GV, Stroyakovskiy D, Gogas H, Levchenko E, de Braud F, Larkin J, Garbe C, Jouary T, Hauschild A, Grob JJ, Sileni VC, Lebbe C, Mandala M (2014). Combined BRAF and MEK inhibition versus BRAF inhibition alone in melanoma. N Engl J Med.

[22] Larkin J, Ascierto PA, Dreno B, Atkinson V, Liszkay G, Maio M, Mandala M, Demidov L, Stroyakovskiy D, Thomas L, L de la Cruz-Merino, Dutriaux C, Garbe C (2014). Combined vemurafenib and cobimetinib in BRAF-mutated melanoma. N Engl J Med.

[23] Jakob JA, Bassett RL, Ng CS, Curry JL, Joseph RW, Alvarado GC, Rohlfs ML, Richard J, Gershenwald JE, Kim KB, Lazar AJ, Hwu P (2012). NRAS mutation status is an independent prognostic factor in metastatic melanoma. Cancer.

[24] Colombino M, Capone M, Lissia A, Cossu A, Rubino C, De Giorgi V, Massi D, Fonsatti E, Staibano S, Nappi O, Pagani E, Casula M, Manca A (2012). BRAF/NRAS mutation frequencies among primary tumors and metastases in patients with melanoma. J Clin Oncol.

[25] Thumar J, Shahbazian D, Aziz SA, Jilaveanu LB, Kluger HM (2014). MEK targeting in N-RAS mutated metastatic melanoma. Mol Cancer.

[26] Ascierto PA, Schadendorf D, Berking C, Agarwala SS, van Herpen CM, Queirolo P, Blank CU, Hauschild A, Beck JT, St-Pierre A, Niazi F, Wandel S, Peters M (2013). MEK162 for patients with advanced melanoma harbouring NRAS or Val600 BRAF mutations: a non-randomised, open-label phase 2 study. Lancet Oncol.

[27] Hughes PE, Rex K, Caenepeel S, Yang Y, Zhang Y, Broome MA, Kha HT, Burgess TL, Amore B, Kaplan-Lefko PJ, Moriguchi J, Werner J, Damore MA (2016). *In Vitro* and *In Vivo* Activity of AMG 337, a Potent and Selective MET Kinase Inhibitor, in MET-Dependent Cancer Models. Mol Cancer Ther.

[28] Boezio AA, Copeland KW, Rex K, B KA, Bauer D, Bellon SF, Boezio C, Broome MA, Choquette D, Coxon A, Dussault I, Hirai S, Lewis R (2016). Discovery of (R)-6-(1-(8-Fluoro-6-(1-methyl-1H-pyrazol-4-yl)-[1,2,4]triazolo[4,3-a]pyridin-3-y l)ethyl)-3-(2-methoxyethoxy)-1,6-naphthyridin-5(6H)-one (AMG 337), a Potent and Selective Inhibitor of MET with High Unbound Target Coverage and Robust In Vivo Antitumor Activity. J Med Chem.

[29] Barrett SD, Bridges AJ, Dudley DT, Saltiel AR, Fergus JH, Flamme CM, Delaney AM, Kaufman M, LePage S, Leopold WR, Przybranowski SA, Sebolt-Leopold J, Van Becelaere K (2008). The discovery of the benzhydroxamate MEK inhibitors CI-1040 and PD 0325901. Bioorg Med Chem Lett.

[30] Norman MH, Andrews KL, Bo YY, Booker SK, Caenepeel S, Cee VJ, D’Angelo ND, Freeman DJ, Herberich BJ, Hong FT, Jackson CL, Jiang J, Lanman BA (2012). Selective class I phosphoinositide 3-kinase inhibitors: optimization of a series of pyridyltriazines leading to the identification of a clinical candidate, AMG 511. J Med Chem.

[31] Gherardi E, Birchmeier W, Birchmeier C, Vande Woude G (2012). Targeting MET in cancer: rationale and progress. Nat Rev Cancer.

[32] Faletto DL, Tsarfaty I, Kmiecik TE, Gonzatti M, Suzuki T, Vande Woude GF (1992). Evidence for non-covalent clusters of the c-met proto-oncogene product. Oncogene.

[33] Avraham R, Yarden Y (2011). Feedback regulation of EGFR signalling: decision making by early and delayed loops. Nat Rev Mol Cell Biol.

[34] Lito P, Pratilas CA, Joseph EW, Tadi M, Halilovic E, Zubrowski M, Huang A, Wong WL, Callahan MK, Merghoub T, Wolchok JD, de Stanchina E, Chandarlapaty S (2012). Relief of profound feedback inhibition of mitogenic signaling by RAF inhibitors attenuates their activity in BRAFV600E melanomas. Cancer Cell.

[35] Carnahan J, Beltran PJ, Babij C, MJ Le Q Rose, Vonderfecht S, Kim JL, Smith AL, Nagapudi K, Broome MA, Fernando M, Kha H, Belmontes B (2010). Selective and potent Raf inhibitors paradoxically stimulate normal cell proliferation and tumor growth. Mol Cancer Ther.

[36] Kugel CH, Aplin AE (2014). Adaptive resistance to RAF inhibitors in melanoma. Pigment Cell Melanoma Res.

[37] Sharma SV, Lee DY, Li B, Quinlan MP, Takahashi F, Maheswaran S, McDermott U, Azizian N, Zou L, Fischbach MA, Wong KK, Brandstetter K, Wittner B (2010). A chromatin-mediated reversible drug-tolerant state in cancer cell subpopulations. Cell.

[38] Smith AL, DeMorin FF, Paras NA, Huang Q, Petkus JK, Doherty EM, Nixey T, Kim JL, Whittington DA, Epstein LF, Lee MR, Rose MJ, Babij C (2009). Selective inhibitors of the mutant B-Raf pathway: discovery of a potent and orally bioavailable aminoisoquinoline. J Med Chem.

[39] Zhao H, Wolfe T, Plewa C, Sheng J, Lee KJ (2012). Scalable single-step affinity purification of rAAV vector using AVB Sepharose yields high purity and high titer vector. Molecular therapy.

