# Supplementary material for: MAPK pathway inhibition induces MET and GAB1 levels, priming BRAF mutant melanoma for rescue by hepatocyte growth factor
Source: Oncotarget. 2017 Jan 27;8(11):17795–809. doi: 10.18632/oncotarget.14855 (PMC5392287; doi:10.18632/oncotarget.14855)
Supplement: Supplementary file 1 [file oncotarget-08-17795-s001.pdf]

MAPK pathway inhibition induces MET and GAB1 levels, priming BRAF mutant melanoma for rescue by hepatocyte growth factor

Supplementary Materials

Cell Lines

BRAF^{V600E} mutant melanoma cell lines A101D, A2058, A375, C32, COLO829, G361, M14, Malme3M, RPMI7951, SH4, SK-MEL-5, SK-MEL-24, and SK-MEL-28 were obtained from American Type Culture Collection. COLO679 was obtained from Sigma-Aldrich, and all remaining BRAF and NRAS mutant melanoma cell lines were obtained from Dr. Antoni Ribas (University of California, Los Angeles). The M-series human melanoma cell lines were established from patient biopsies under UCLA IRB 11-003254, as previously described [refs. J. N. Sondergaard et al. J Translational Med 2010, 8: 39 and Nazarian et al. Nature 2010].

Generation of Tet-HGF-G361 cell line

The Tet-HGF-G361 cell line was generated using the ViraPower™ HiPerform™ T-REx™ Gateway® Expression System from Life Technologies. G361 parental cells were transduced with lentivirus that constitutively express high levels of the Tet repressor under control of the cytomegalovirus (CMV) promoter. Following selection with 500 µg/mL neomycin, cells were transduced with lentivirus expressing human HGF under control of the Tet-regulated, hybrid CMV/TO promoter. Tet-HGF-G361 cells were selected with 5 µg/mL blasticidin.

Flow cytometry

Cell surface expression of MET was measured with a mouse anti-human MET PE-conjugated antibody (R&D Systems). A mouse Immunoglobulin G PE-conjugated antibody (SouthernBiotech) was used as an isotype control. Antibodies were diluted (250 ng/mL) for staining reactions with cultured cells (1×10^6 cells/mL) in fluorescence-activated cell sorting buffer (phosphate-buffered saline [PBS] + 1% fetal calf serum + 0.01% NaN_3). Samples were run on an LSR-II flow cytometer (BD Biosciences), counting 20,000 events/treatment condition. Analysis was performed using FCS Express Research v4 software (*De Novo* Software).

Immunoblot analysis

Samples were prepared at normalized concentrations in NuPAGE® LDS Sample Buffer per manufacturer protocol, electrophoresed on 10% Bis-Tris NuPAGE® gels (Life Technologies), transferred to 0.45 µm polyvinylidene fluoride membranes, and blocked in 5% milk/tris-buffered saline with Tween 20 (TBST). Membranes were incubated with total or phospho-specific antibodies at 1:1000 dilutions (beta-actin at 1:5000) in either 5% milk/TBST (total protein) or 5% bovine serum albumin/TBST (phosphorylated protein) overnight at 4°C. Proteins were detected with horseradish peroxidase–conjugated antibodies, SuperSignal® West Pico or Dura Extended Duration Substrate (Thermo Fisher Scientific), and the ChemiDoc™ Imaging System (Bio-Rad).

Electro-chemiluminescence immuno assay

Standard curves were performed using control cell lysates containing high levels of target protein (11-step, four-fold dilution series). Unknown samples were profiled at 25 µg/well. Electrochemiluminescent signals for unknown samples were confirmed to be in the linear range of the standard curve; relative concentrations of unknown samples were calculated by MSD workbench software. All samples were run in duplicate.

Supplementary Table 1: Clinical data for patient derived BRAF and NRAS mutant melanoma cell lines. see Supplementary_Table_1

Supplementary Table 2: Strength of HGF-Mediated rescue from vemurafenib treatment in a panel of BRAF^{V600E} mutant melanoma cell lines

	BRAF Mutation	Fold Rescue*	HGF Rescue Status
G361	V600E	4.2	Strong
COLO679	V600E	3.1	Strong
SK-MEL-5	V600E	2.2	Moderate
SK-MEL-24	V600E	1.4	Moderate
A101D	V600E	1.2	Moderate
A375	V600E	1.0	No rescue
M14	V600E	0.9	No rescue

HGF = hepatocyte growth factor.

$$\text{*Fold HGF rescue} = \frac{\text{vemurafenib (3 μM) + HGF (33 ng/mL)}}{\text{vemurafenib (3 μM)}} \text{ 72 hour viability assay.}$$

A

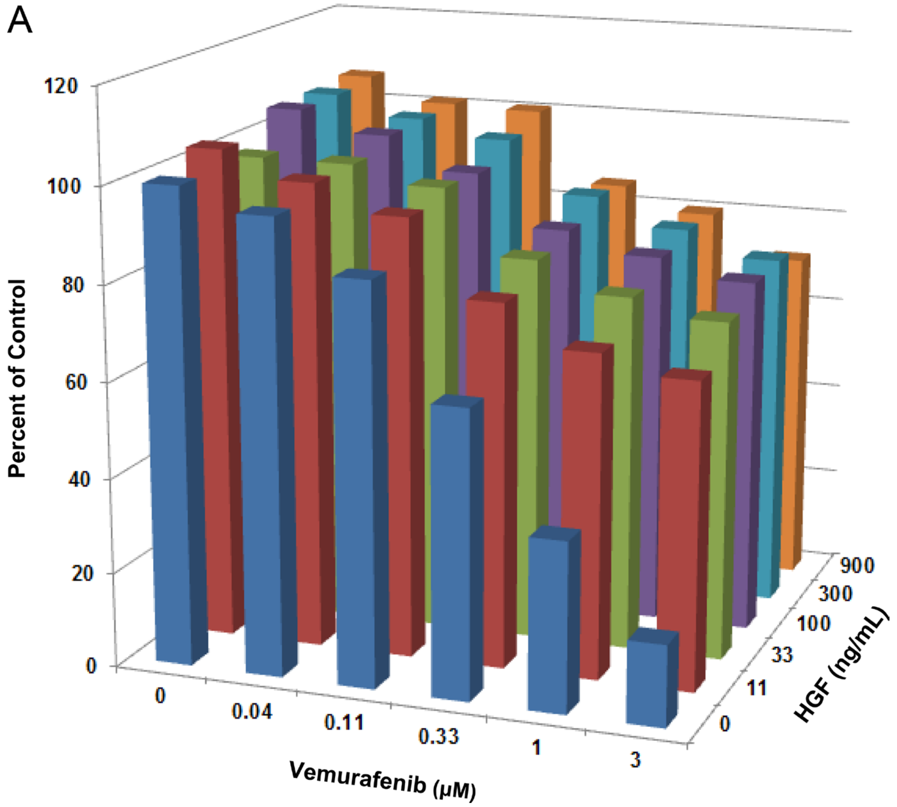

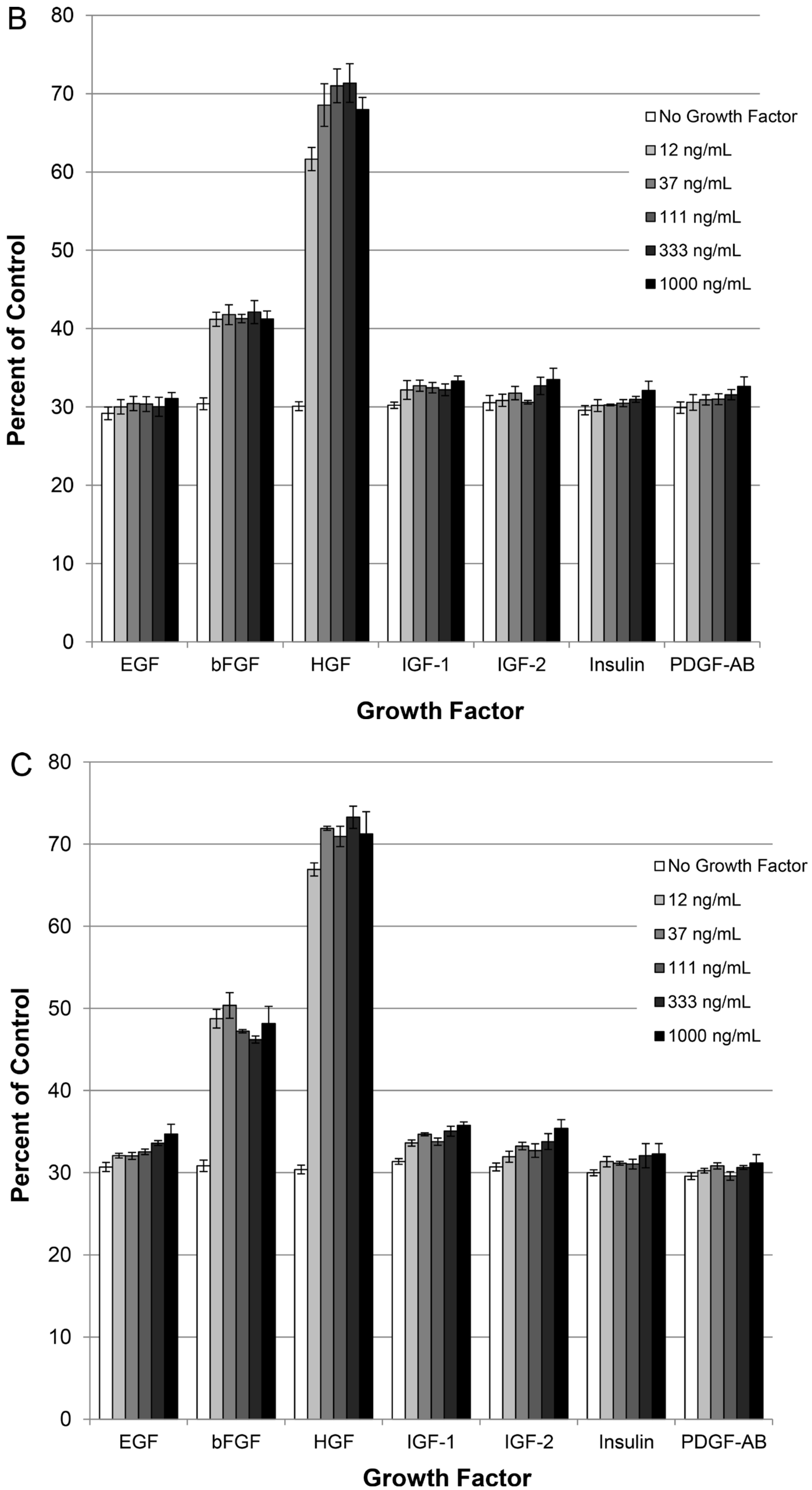

Supplementary Figure 1: HGF treatment rescues BRAF^{V600E} mutant melanoma cells from the effects of BRAF inhibition. (**A**) G361 cells were cultured in the presence of a serial dilution matrix of vemurafenib and HGF for 72 hours. Effects on viability were quantified (ATP concentration) and normalized to the no-treatment control. (**B**) COLO679 and (**C**) G361 BRAF^{V600E} mutant melanoma cells were cultured in the presence of vemurafenib (2 μM) and one of seven growth factors for 72 hours. Effects on viability were quantified (ATP concentration) and normalized to the no-growth factor control. Bars represent SD across replicates (*n* = 4).

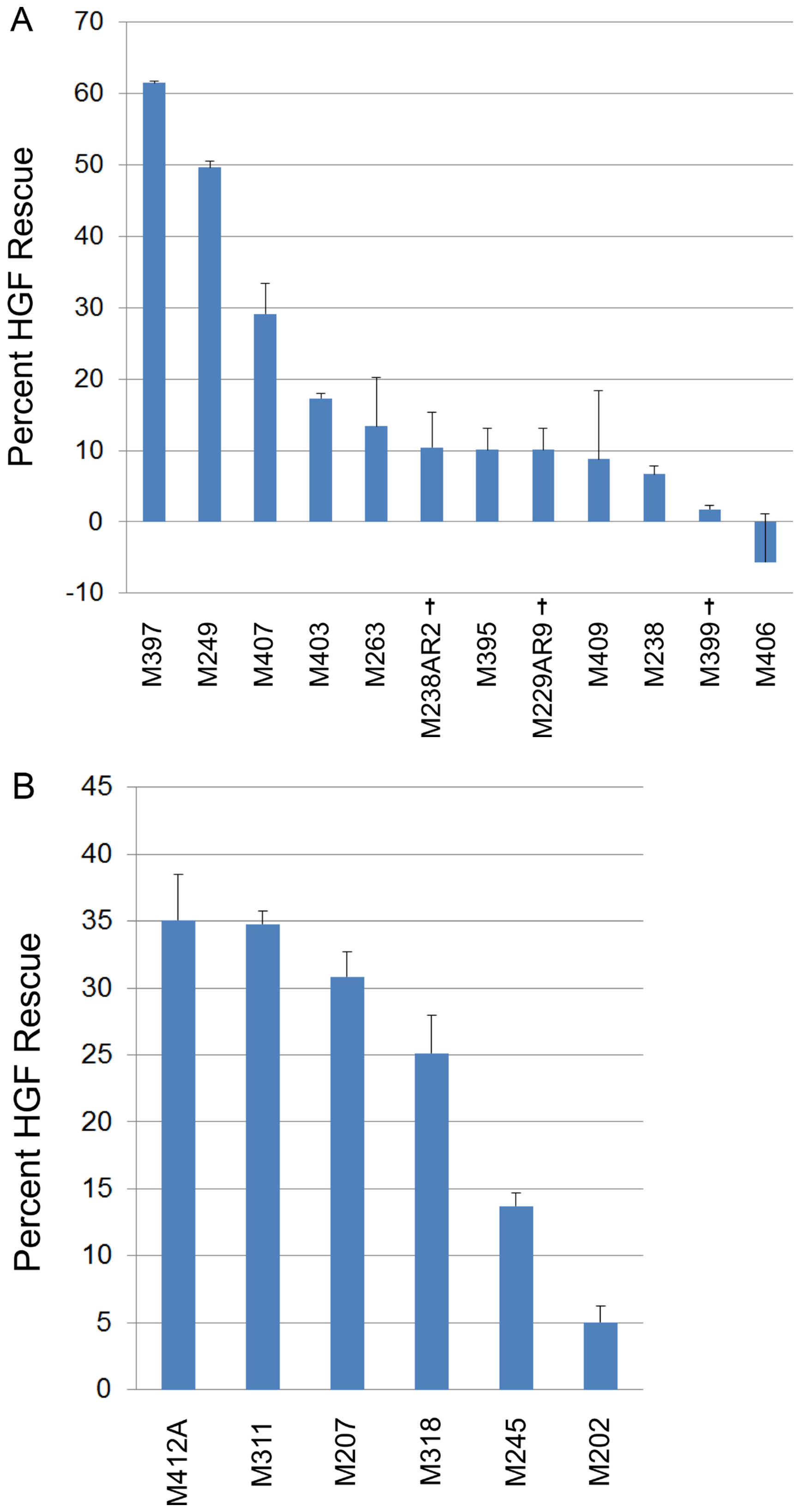

Supplementary Figure 2: HGF treatment rescues patient-derived BRAF mutant and NRAS mutant melanoma cell lines from the effects of BRAF or MEK inhibition respectively. (**A**) Patient derived BRAF mutant melanoma cell lines were cultured in the presence of 800 nM or 3 µM (†) dabrafenib and 333 ng/mL HGF for 72 hours. Effects on viability were quantified (ATP concentration) and reported as percent HGF rescue relative to drug treatment alone. (**B**) Patient derived NRAS mutant melanoma cell lines cultured in the presence of 30 nM trametinib and 333 ng/mL HGF for 72 hours. Effects on viability were quantified (ATP concentration) and reported as percent HGF rescue relative to drug treatment alone. Bars represent standard deviation across replicates (n = 2).

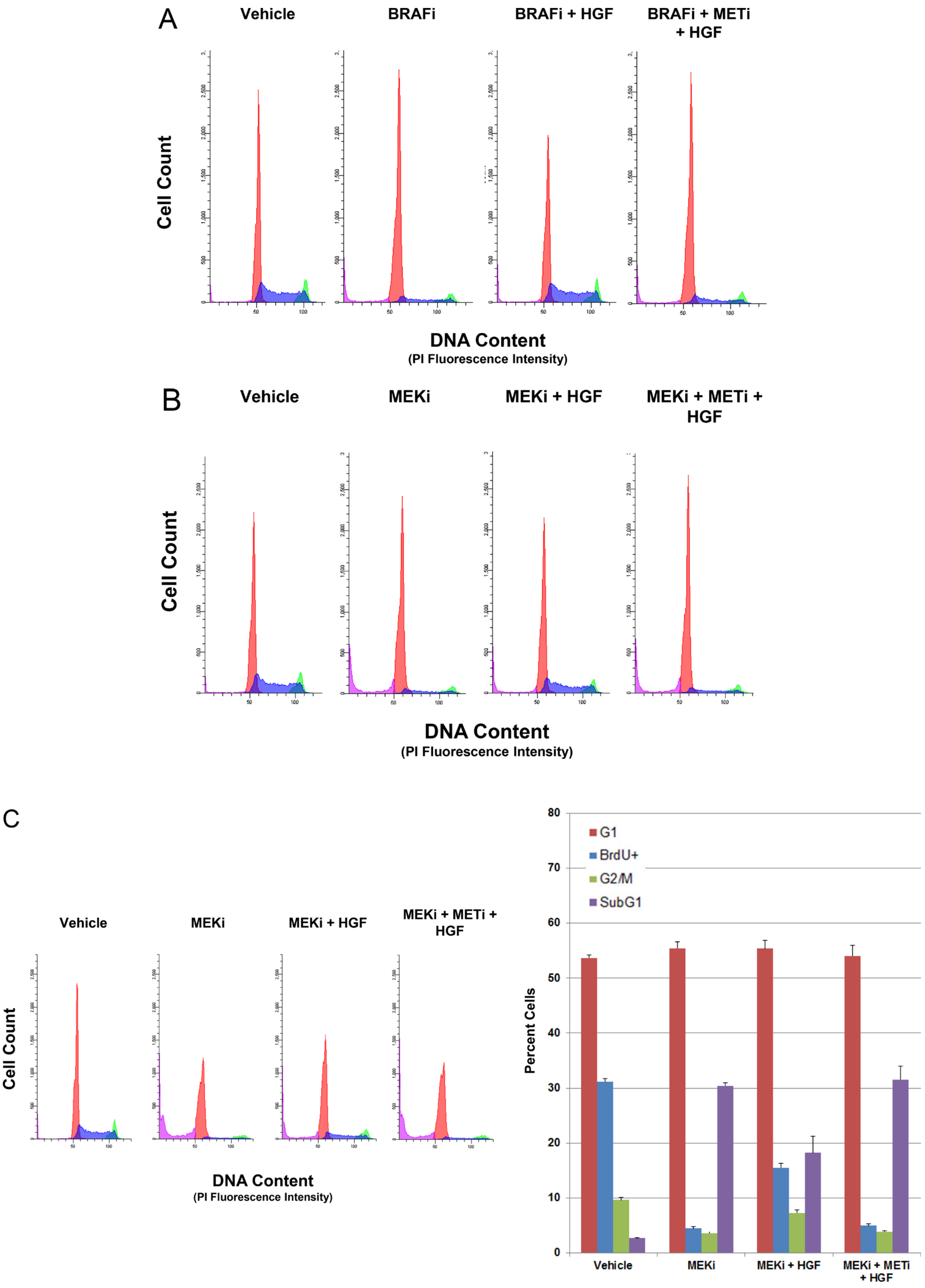

Supplementary Figure 3: MET inhibition attenuates HGF rescue of BRAF and MEK inhibitors in BRAF^{V600E} mutant melanoma cell line G361. (**A**) Cells were cultured in the presence of vehicle, vemurafenib (2 µM), vemurafenib + HGF (100 ng/mL), or vemurafenib + HGF + Compound 20 (100 nM) for 48 hours. BrdU labeling reagent was added to cells for the final two hours of the treatment period. Cells were harvested, fixed, permeabilized, stained and analyzed by flow cytometry. Figure shows corresponding ploidy histograms for results summarized in Figure 3B. (**B**) As described in S3A but with PD0325901 (200 nM) substituted for vemurafenib. Figure shows corresponding ploidy histograms for results summarized in Figure 3B. (**C**) As described in S3A but with PD0325901 (1 µM) substituted for vemurafenib.

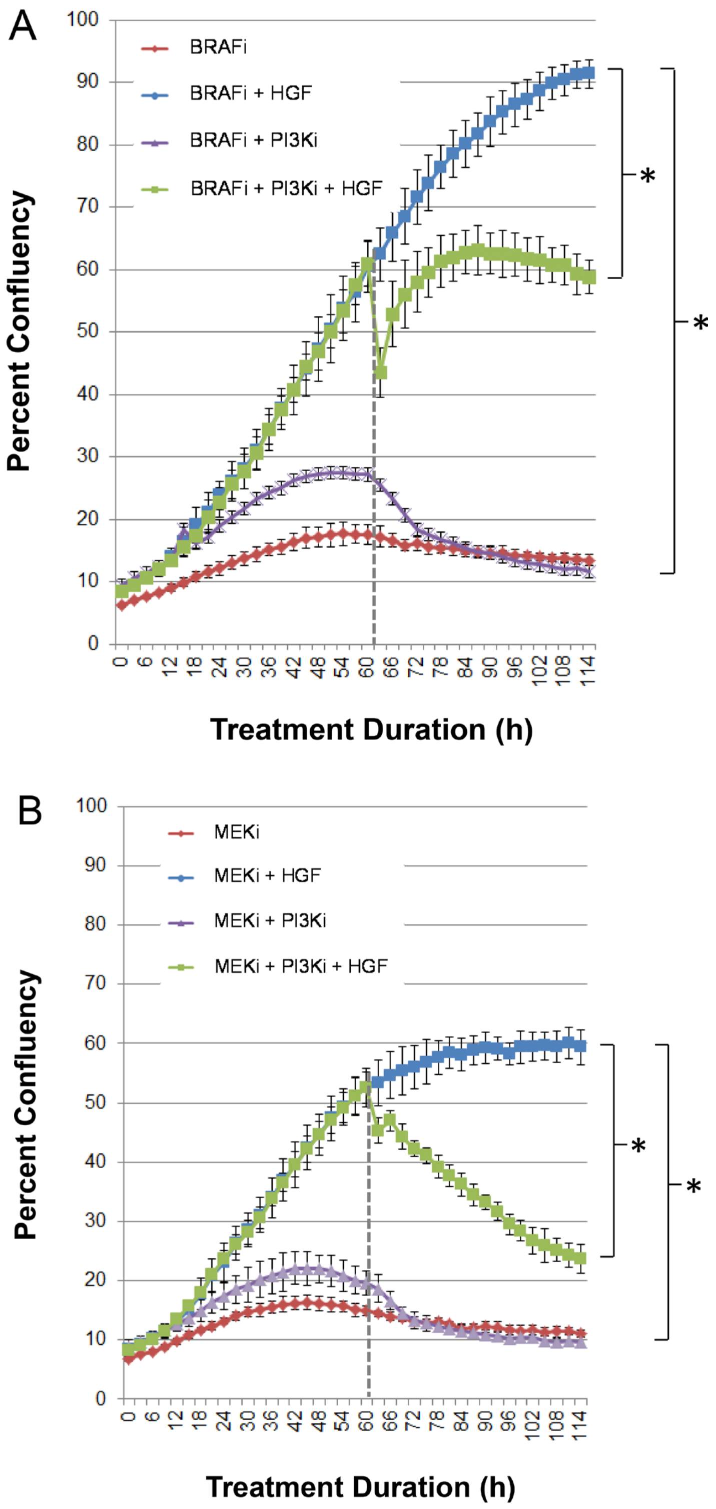

Supplementary Figure 4: PI3K inhibition attenuates HGF rescue of BRAF and MEK inhibitors. (**A**) G361 $BRAF^{V600E}$ mutant melanoma cells were cultured in the presence of vemurafenib (2 μM) or vemurafenib + HGF (500 ng/mL). AMG 511 (3 μM) was added to indicated conditions at the 60-hour time point (broken line). Cells were imaged every 3 hours, tracking changes in confluency as a measure of effect on proliferation. Bars represent SD across replicate wells ($n = 3$). Statistical analysis performed using Student two-tailed t test; $^*P < 0.01$. (**B**) As described in Supplementary Figure 4A but with PD0325901 (1 μM). Statistical analysis performed using Student two-tailed t test; $^*P < 0.01$.

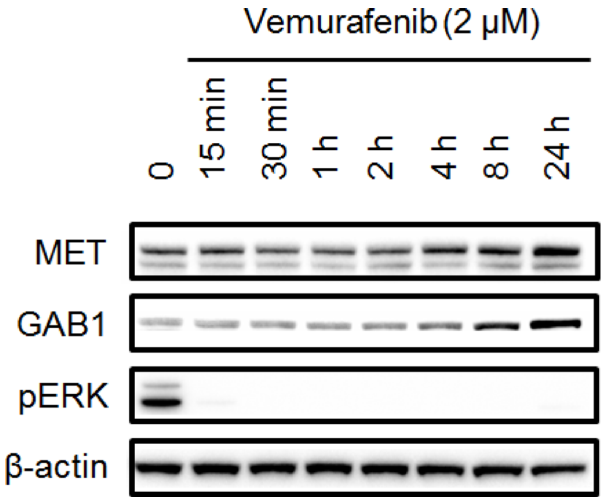

Supplementary Figure 5: Delay in MET and GAB1 protein induction following MAPK pathway inhibition (pERK) suggests the underlying mechanism may be related to changes in transcription. Immunoblot analysis of G361 cells following a vemurafenib (2 µM) time course.

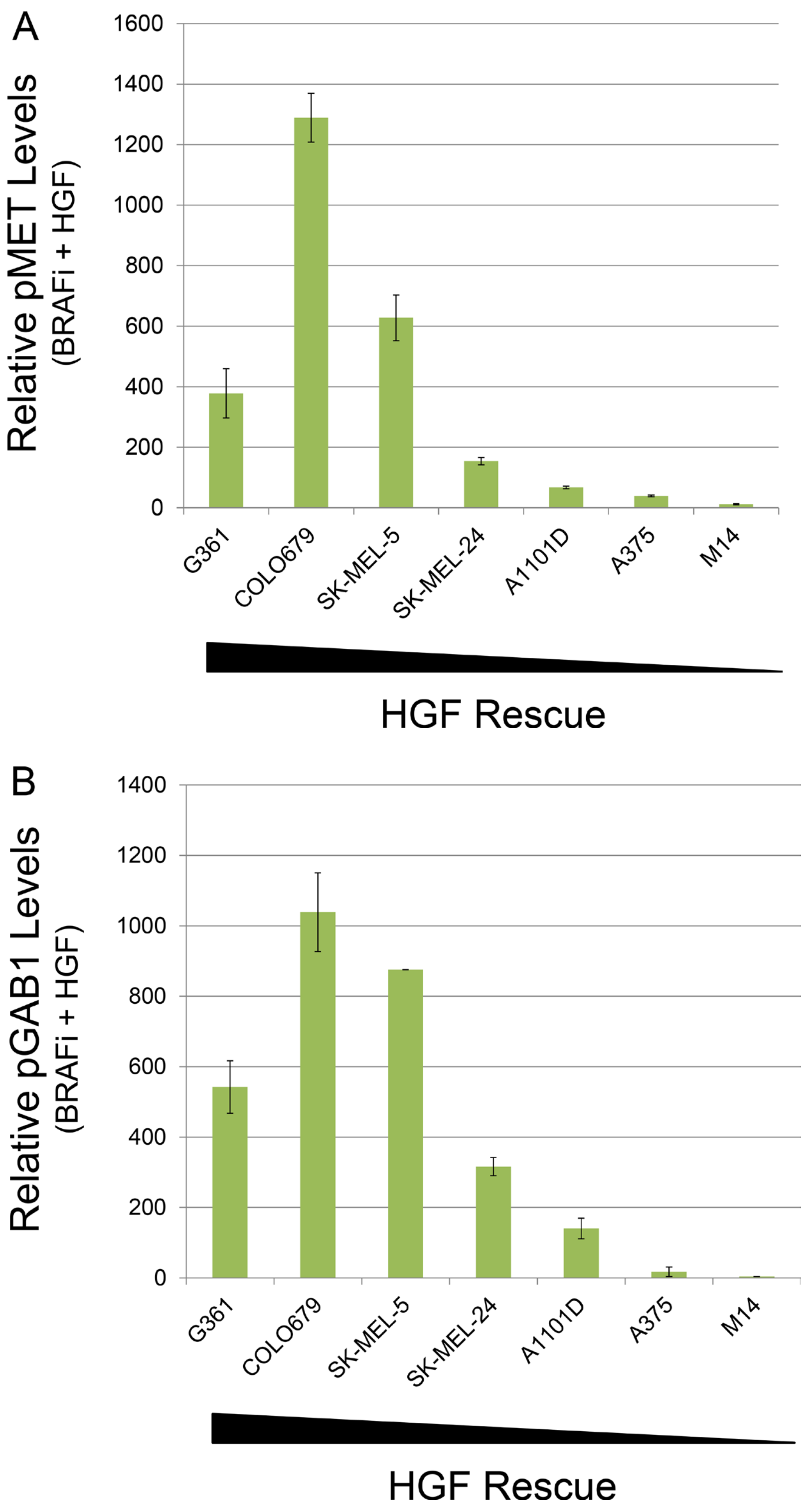

Supplementary Figure 6: pMET (Y1349) and pGAB1 (Y627) levels following HGF rescue of BRAFi treatment predict for strength of HGF rescue in BRAF^{V600E} mutant melanoma cell lines. (**A**) Seven BRAFV600E mutant melanoma cell lines exhibiting varying degrees of HGF-mediated rescue (cell lines rank order left to right based on strength of HGF rescue) were treated with vemurafenib (2 µM) + HGF (100 ng/mL) for 24 hours. pMET (Y1349) levels were measured using Meso Scale Discovery assays. (**B**) As described in Supplementary Figure 4A, but measuring pGAB1 (Y627) levels. Reported pMET and pGAB1 values are not normalized to total MET and GAB1 protein values.

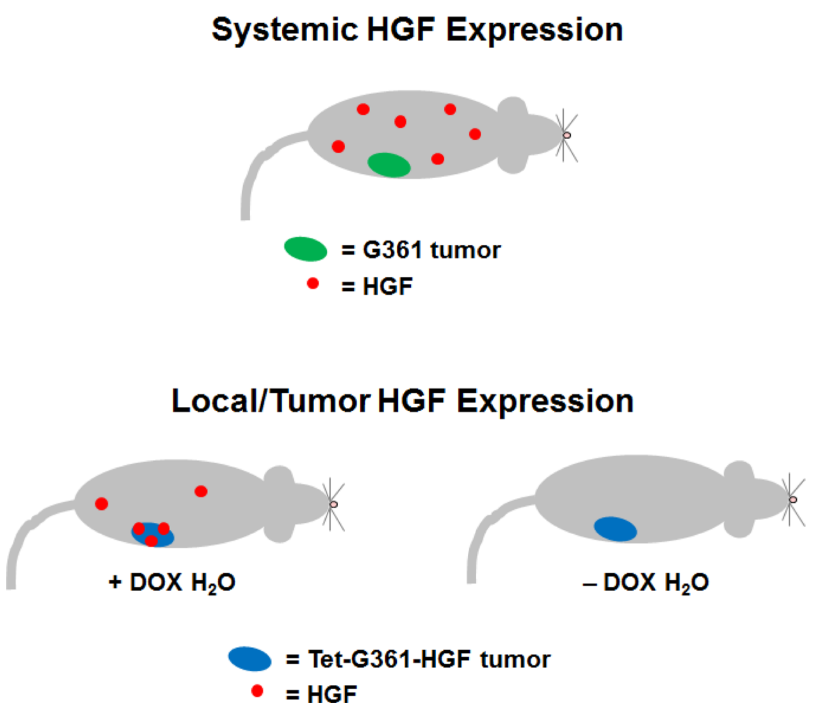

Supplementary Figure 7: Diagram of *in vivo* models used to address requirement for local/tumor vs systemic HGF expression in BRAF inhibitor resistance.

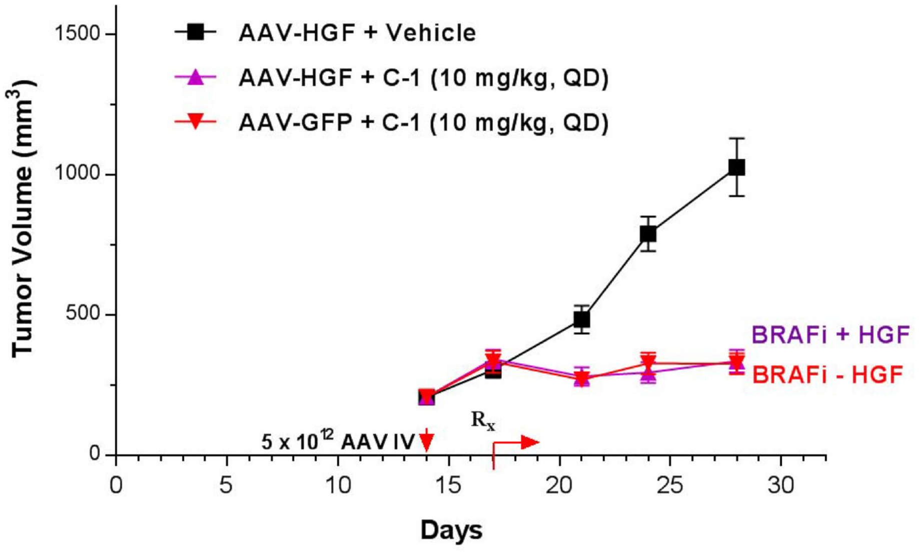

Supplementary Figure 8: Systemic HGF expression via high-dose AAV-HGF failed to rescue G361 xenografts from the growth inhibitory effects of C-1. Athymic nude mice bearing $BRAF^{V600E}$ mutant melanoma G361 tumor xenografts were treated intravenously with recombinant AAV vector containing human HGF (AAV-HGF) or GFP (AAV-GFP; 5×10^{12} viral particles/mouse). Three days post-administration, mice were treated with C-1 (10 mg/kg QD) or vehicle. Tumor volumes were recorded twice weekly (mean ± SEM).

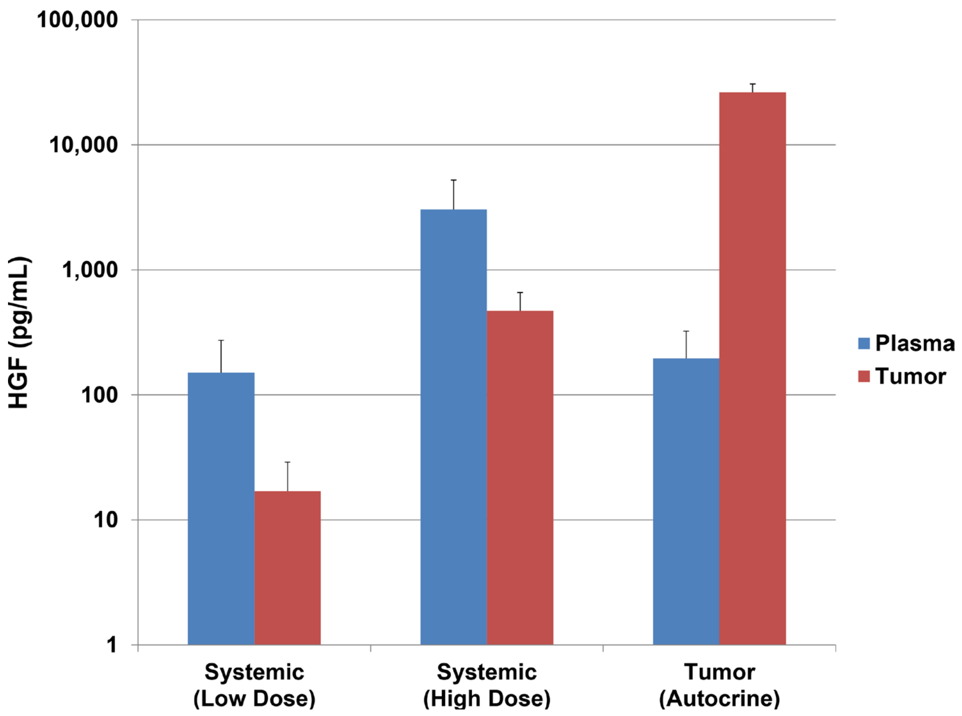

Supplementary Figure 9: Highest plasma HGF levels were observed in the high-dose AAV-HGF animals, whereas the highest tumor HGF levels were observed in the Tet-HGF model. Terminal plasma and tumor HGF levels were measured in triplicate using the Meso Scale Discovery HGF assay.

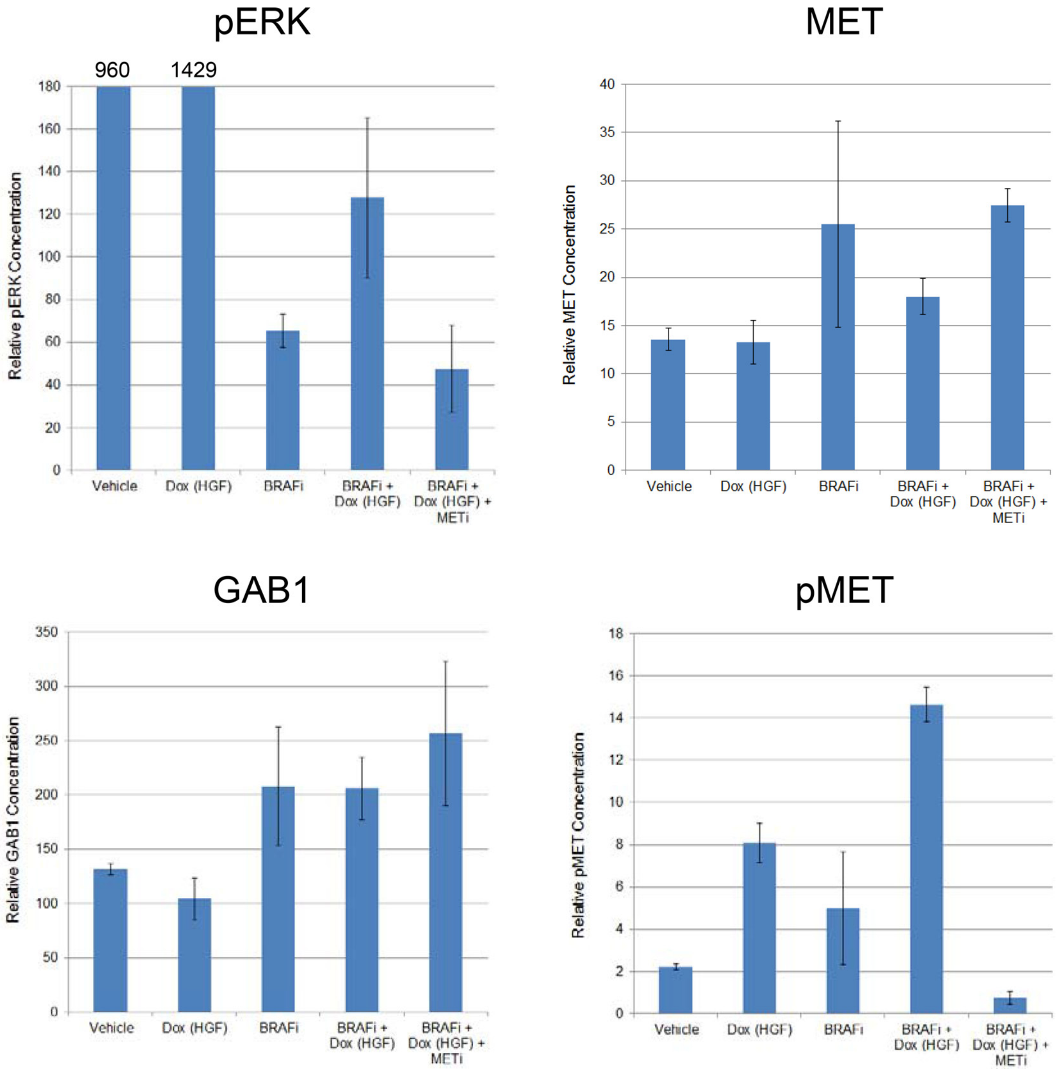

Supplementary Figure 10: Induction of MET and GAB1 protein levels following BRAF inhibitor treatment primes BRAF^{V600E} mutant melanoma xenografts for rescue by HGF. Meso Scale Discovery assay analysis of indicated signaling proteins from dedicated pharmacodynamic study of Tet-HGF-G361 tumor xenograft samples collected 6 hours after final drug treatment.

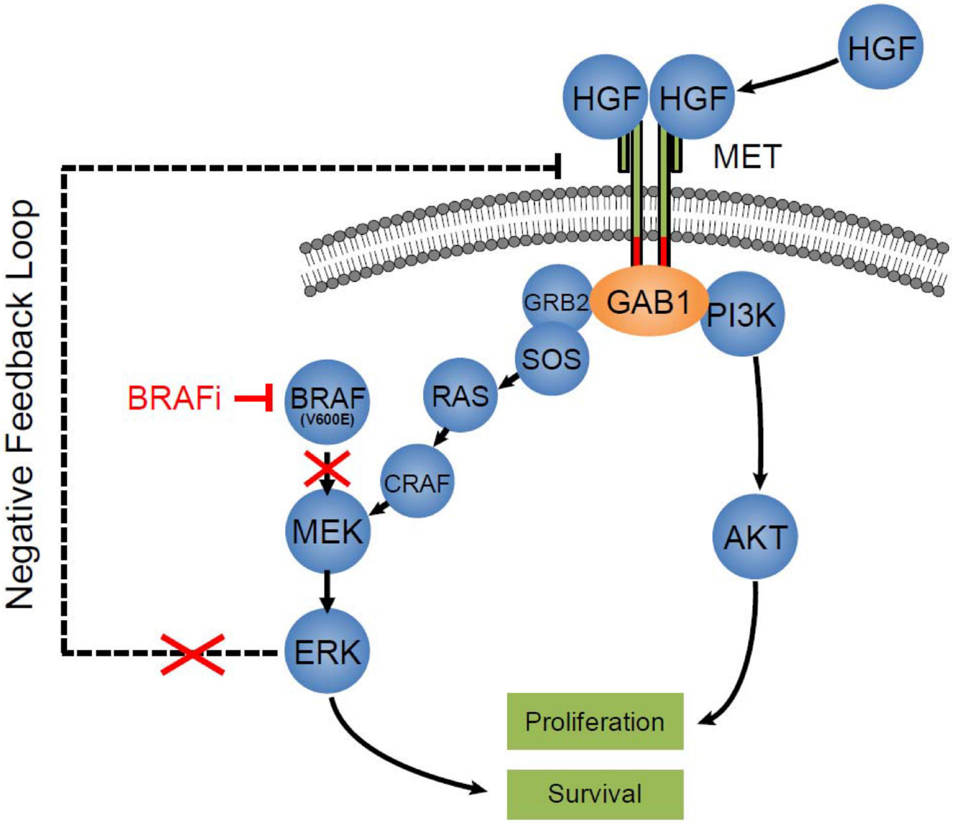

Supplementary Figure 11: Model of HGF-mediated resistance to MAPK pathway inhibition in BRAF mutant melanoma. Oncogenic BRAF drives ERK-dependent feedback, repressing RTK (MET) and adaptor protein (GAB1) signaling. BRAF inhibition relieves these negative feedback loops, resulting in elevated MET and GAB1, priming signaling for HGF-mediated rescue.
